# Humoral IgG1 responses to tumor antigens underpin clinical outcomes in immune checkpoint blockade

**DOI:** 10.1038/s41591-025-04177-6

**Published:** 2026-01-27

**Authors:** Edgar Gonzalez-Kozlova, Robert Sweeney, Igor Figueiredo, Kevin Tuballes, Sinem Ozbey, Pauline Hamon, Matthew D. Park, Giorgio Ioannou, Yohei Nose, Ruiwei Guo, Paula Restrepo, Mark Buckup, Vladimir Roudko, Clotilde Hennequin, Jessica Le Berichel, Nicholas Venturini, Laszlo Halasz, Leanna Troncoso, Alexandra Tabachnikova, Christie Chang, Amanda Reid, Haley Brown, Theodore Chin, Rafael Cabal, Raphaël Mattiuz, Shingo Eikawa, Diane Marie Del Valle, Tina Ruth Gonsalves, Nelson M. LaMarche, Hajra Jamal, Alona Lansky, Nancy Yi, Daniella Nelson, Jarod Morgenroth-Rebin, Raphael Merand, Bryan Villagomez, Darwin D’Souza, Emir Radkevich, Kai Nie, Zhihong Chen, Yasuko Tada, Hiroyoshi Nishikawa, Stephen C. Ward, Maria Isabel Fiel, Rachel Brody, Parissa Tabrizian, Ganesh Gunasekaran, Alice O. Kamphorst, Noah Cohen, Maria Curotto de Lafaille, Olivia Hapanowicz, Natalie Lucas, Kathy Wu, Nicola James, John C. Lin, Gavin Thurston, Myron Schwartz, Nathalie Fiaschi, Seunghee Kim-Schulze, Miriam Merad, Thomas U. Marron, Sacha Gnjatic

**Affiliations:** 1 Department of Immunology and Immunotherapy, Icahn School of Medicine at Mount Sinai, New York, NY USA; 2 Marc and Jennifer Lipschultz Precision Immunology Institute, Icahn School of Medicine at Mount Sinai, New York, NY USA; 3 Tisch Cancer Institute, Icahn School of Medicine at Mount Sinai, New York, NY USA; 4 Human Immune Monitoring Center, Icahn School of Medicine at Mount Sinai, New York, NY USA; 5 Division of Cancer Immunology, Research Institute EPOC, National Cancer Center, Tokyo and Chiba, Japan; 6 Regeneron Pharmaceuticals, Inc., Tarrytown, NY USA

**Keywords:** Immunology, Statistical methods

## Abstract

Tumor-infiltrating T cells have been the primary focus of cancer immunotherapy; however, accumulating evidence points to a critical role for B cells and plasma cells in shaping responses to immune checkpoint blockade. In this study, we investigated the humoral immune response in 38 patients with hepatocellular carcinoma treated with neoadjuvant anti-programmed cell death protein 1 (PD-1) therapy. In responders, defined by more than 50% tumor necrosis, we observed on-treatment enrichment of clonally expanded IgG1^+^ plasma cells within the tumor. Clonal tracking revealed that anti-PD-1 treatment expanded preexisting B cell clones associated with favorable clinical outcomes. Moreover, serum from responders contained IgG1 antibodies specific to cancer/testis antigens, including NY-ESO-1, and these humoral responses were linked to tumor-reactive T cell activity. We independently validated these findings across seven additional cohorts, encompassing single-cell and bulk sequencing data from 500 patients, spatial transcriptomics from seven patients and survival analyses from 1,582 patients. Our findings apply to recently approved treatments, such as PD-1 and vascular endothelial growth factor A (VEGF-A) blockade, but not to chemotherapy alone, suggesting broad relevance to individuals treated with immunotherapy. Collectively, our results demonstrate that PD-1 blockade induces tumor-specific IgG1^+^ plasma cell responses that complement cellular immunity and contribute to clinical benefit, underscoring a coordinated humoral−cellular axis in effective antitumor immunity.

## Abstract (web summary)

Transcriptomic and clinical data analyses from multiple cohorts of patients with cancer receiving immunotherapy find that PD-1 blockade potentiates tumor-specific IgG1 plasma cell responses that complement cellular immunity and contribute to clinical benefit.

## Main

Immune checkpoint blockade (ICB) has become the backbone in the treatment of numerous types of cancer, although there remain major gaps in mechanistic understanding of what leads to clinical response^1,2^. ICB primarily targets inhibitory pathways, such as PD-1/programmed death ligand 1 (PD-L1) and cytotoxic T lymphocyte antigen 4 (CTLA-4), that regulate T cell function, thereby enhancing T cell activation, infiltration, cytotoxicity and antitumor immune responses^3,4^. In addition to T cells, ICB also has the potential to modulate other lymphoid and myeloid lineages, including B cells, natural killer (NK) cells and dendritic cells. A robust tumor immune microenvironment response requires the coordination of several immune cell types to activate cytotoxic tumor-infiltrating lymphocytes (TILs)^5^. Previously, we described intratumoral niches containing mature dendritic cells and CXCL13^+^ helper T cells, leading to a coordinated clonal expansion of granzyme K^+^ and PD-1^+^ effector-like CD8^+^ T cells in ICB responders with early-stage hepatocellular carcinoma (HCC)^6^.

In several cancers, infiltrating plasma cells (PCs) and B cells carry strong prognostic significance and have emerged as potential predictors of response to immune checkpoint inhibitors^7^^,8^^,9^. Moreover, they can perform a variety of functions, including antigen presentation and antibody production, which enable them to support both T cell responses and innate mechanisms such as complement activation and opsonization of cancer antigens^7^. Tumor-associated B cells have been shown to play a crucial role in melanoma inflammation and have been associated with response to ICB therapy^10^. Intratumoral B cells and PCs identified by single-cell RNA sequencing (scRNA-seq) in non-small-cell lung cancer (NSCLC) showed predictive association with overall survival to PD-L1 blockade, independently of intratumoral CD8^+^ T cells and PD-L1 expression^11^. Additionally, these cells can also be present in tertiary lymphoid structures (TLSs), which may contribute to their differentiation into immunoglobulin-secreting plasma cells^11,12^. These TLSs, particularly those with mature organization with T cells and PCs surrounding germinal center class-switching B cells, have been associated with clinical benefit in multiple studies^13^.

Although a predictive association between PCs and overall survival in patients treated with anti-PD-1 or anti-PD-L1 (PD-(L)1) therapy blockade has been established, the underlying intricate mechanisms (clonal composition and dynamics, isotype and subclass usage and specificity) driving this association are poorly understood, and no study to date has demonstrated the ability of PD-(L)1 blockade to induce or potentiate humoral antitumor immunity^7,14^. Furthermore, B cells represent a highly heterogenous and diverse population, and the specific subset of B cells or PCs that might be most important for effective antitumor immunity has not been clearly elucidated.

In the present study, we investigated B cells and PCs during ICB responses with neoadjuvant anti-PD-1 therapy, with or without radiation, in HCC. We used bulk, single-cell and B cell receptor (BCR) RNA sequencing to assess longitudinal dynamics and isotype and clonal expansion in tumor and normal tissues as well as lymph nodes. Responders showed tumor-enriched IgG1 class switching, PC differentiation and clonal expansion after ICB, whereas non-responders accumulated dysfunctional memory B cells. The findings were validated in independent cohorts treated with PD-1, PD-L1, CTLA-4 or VEGF-A blockade. Baseline and tumor-enriched IgG1 PCs correlated with clinical response, and elevated circulating IgGs against cancer/testis antigens (CTAs) suggested humoral antitumor immunity in responders after ICB^15–17^.

## Results

### PCs are enriched in clinical responders to PD-1 blockade

**Fig. 1:**
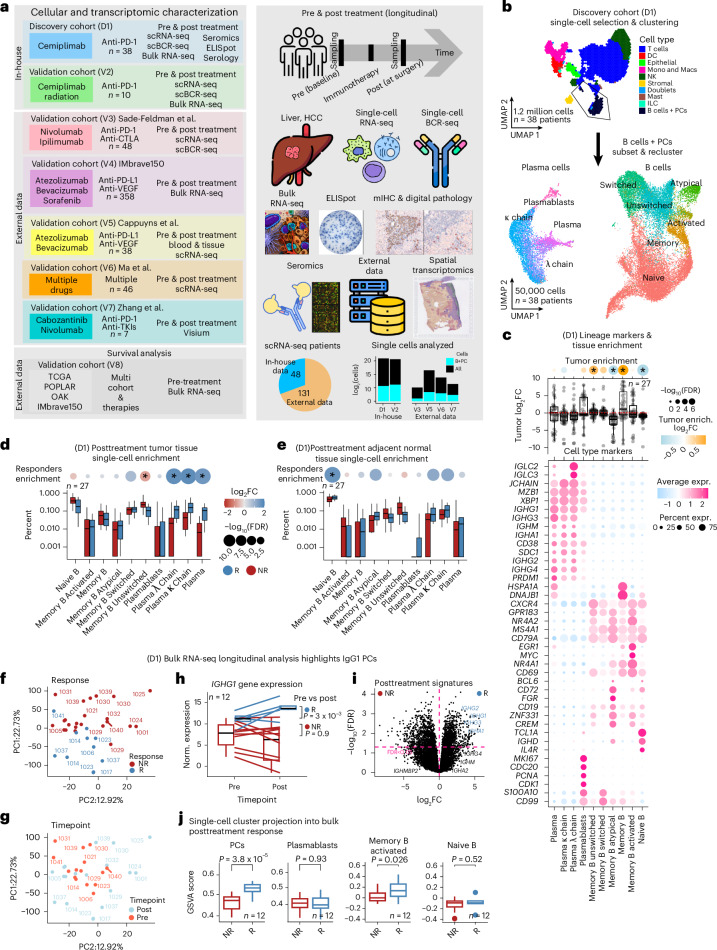
Discovery cohort and the establishment of a plasma IgG1 isotype. **a**, Overview of cohorts analyzed in this study. The discovery cohort (D1) consisted of 38 patients (27 patients with HCC treated with anti-PD-1 and 11 untreated patients with HCC). Seven additional validation cohorts were included. Across all cohorts (D1 and V2), data types encompassed pretreatment and posttreatment samples from 48 in-house and 131 external patients, including bulk and single-cell RNA-seq, BCR-seq, mIHC, spatial transcriptomics, seromics (autoantibody panel), ELISpot and ELISAs. Certain validation cohorts included additional therapeutic contexts—for example, V7 included cabozantinib, a multi-tyrosine kinase inhibitor (TKI). **b**, Uniform manifold approximation and projection (UMAP) plots showing the integrated analysis of 1.2 million cells from 38 patients and the reclustering of 50,000 B cells and PCs. Clusters are colored by cell type/state. Annotation was based on canonical B cell and PC markers together with differentially expressed genes. **c**, Top, tumor enrichment scores for 27 patients, comparing tumor versus adjacent normal tissue using Wilcoxon rank tests with FDR correction. Box plots show medians, interquartile ranges (IQRs) (Q1–Q3), whiskers (≤1.5× IQR) and outliers. Bottom, dot plot of canonical and top differentially expressed genes per cluster, identified by Wilcoxon tests. The adjacent panel shows enrichment of each cluster in normal (light blue) versus tumor (orange); circle size represents statistical significance. **d**,**e**, Box plots (as defined above) showing tissue-specific enrichment of clusters between responders (R; dark blue, *n* = 8) and non-responders (NR; dark red, *n* = 19). Proportions were estimated using Dirichlet regression; log-transformed fold change (log_2_FC) significance was assessed using log-likelihood tests and Benjamini−Hochberg-adjusted *P* values. Color indicates log_2_FC; circle size reflects adjusted *P* value. The *y* axis indicates percent of cells. **f**,**g**, Principal component analysis of bulk RNA-seq showing variance explained by each principal component and sample separation in PC1 and PC2, colored by response category and timepoint (pre/post). **h**, Box plot showing baseline IgG1 expression, higher in responders (*n* = 4) than in non-responders (*n* = 8), with increases after treatment in responders (*P* = 3 × 10^−3^, left-sided Wilcoxon test) and no significant change in non-responders (*P* = 0.8). **i**, Volcano plot showing differential expression between responders and non-responders on-treatment (*x* axis: log_2_FC; *y* axis: –log_10_*P* value). **j**, GSVA scoring of four single-cell-derived signatures projected into bulk showing increased PC signature after treatment in responders (*P* < 0.05, two-sided Wilcoxon). Data represent 12 patients (4 responders and 8 non-responders). DC, dendritic cell; GSVA, gene set variation analysis; ILC, innate lymphoid cell; PC1/2, principal component 1/2.

To test the hypothesis that B cells or PCs have a key role during immunotherapy and treatment response, we examined specimens from eight sets of independent clinical trial cohorts: two newly generated datasets from our own investigator-initiated trials in patients with HCC treated with neoadjuvant PD-1 blockade, with or without radiation, for discovery and validation, respectively. We also analyzed published datasets of a combination of PD-1, PD-L1, CTLA-4 and VEGF-A blockade, including large cohorts such as IMbrave150 and The Cancer Genome Atlas (TCGA) (Fig. 1a). No sex-associated differences were observed in any of the analyzed outcomes, consistent with the balanced representation of male and female patients in the cohort.

**Extended Data Fig. 1:**
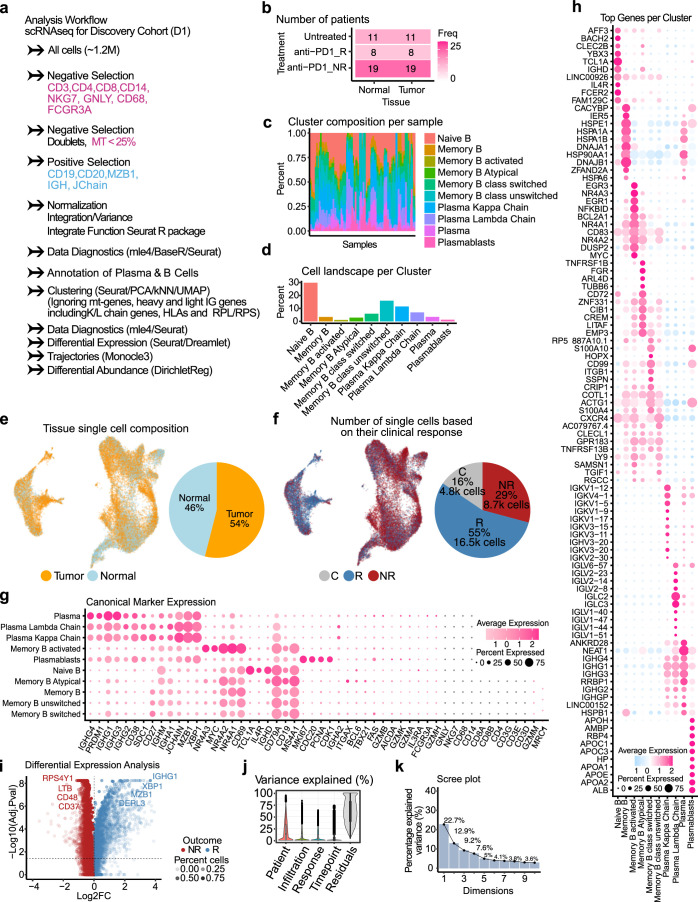
Complementary single cell analysis. **a**. Schematics of the workflow for downstream of cell ranger single cell analysis. **b**. Table showing the number of patients without treatment, with and without treatment stratified in adjacent normal and tumor tissue. **c**. Stacked bar plot showing the cluster composition per sample. **d**. Bar plot showing the landscape of cells per cluster. **e**, **f**. UMAP and pie chart showing that 54% of cells corresponded to tumor tissue and 29% of cells belong to patients who responded to therapy, respectively. **g**. Dot plot showing the percent expressed and average expression levels of canonical genes associated with Plasma and B Cells. In parallel, we show the absence of gene expression associated to non-B/Plasma cells such as *CD3*, *CD4*, *CD8* and similar. **h**. Top 10 most expressed genes per cluster. **i**. Volcano plot showing the significant genes for differential expression between non-responders (NR) in dark red and responders (R) in dark blue. Differential expression was calculated using a two-sided moderate t test through mixed effect models. The top 4 genes based on Log2FC, and percent expressed cells (transparency) are labeled. **j**. Bulk RNAseq HCC cohort, violin plot showing the percent variance explained associated with patient, infiltration, response and timepoint, ordered from most to least variance explained. **k**. Principal component analysis of HCC bulk RNAseq data showing the variance explained for each component.

First, we profiled approximately 30,000 B cells and PCs derived from 1.2 million single-cell transcriptomes of the tumor microenvironment, uninvolved adjacent liver and the draining lymph nodes resected from 38 patients with early-stage HCC of the discovery cohort^18^. Of these, 27 patients received neoadjuvant ICB in the form of an anti-PD-1 blocking antibody, and 11 were untreated controls (Extended Data Fig. 1a,b). We resolved six distinct B cell states (naive, memory, activated, class-switched, class-unswitched and atypical) alongside plasmablasts and three PC populations with discrete transcriptional programs (Fig. 1b). Naive B cells were characterized by the expression of *IGHD* and *CD19* and were decreased in tumor compared to adjacent tissue (false discovery rate (FDR) < 0.05); memory B cells showed *MYC*, *CD69* and *NR4A1* associated with an activation phenotype and with tumor enrichment, whereas PCs were characterized by *MZB1* and *JCHAIN* (Fig. 1c). Naive B cells dominated the B cell fraction of the immune cells across the tissue compartments, followed by memory B cells, relative to total B cells and PCs (Extended Data Fig. 1c–f). As expected, canonical and cluster-specific genes were expressed in more than 70% of cells (Extended Data Fig. 1g,h). Pathological responses to neoadjuvant PD-1 were defined as more than 50% necrosis of tumor at the time of surgery, followed by differential cell abundance showing an enrichment of all PC phenotypes in the tumor among ICB responders (FDR < 0.05), which was not as pronounced in adjacent uninvolved liver (Fig. 1d,e). Conversely, non-responders were enriched in unswitched memory B cells in tumor (FDR < 0.05) but less so in normal tissues (Fig. 1d,e).

### Preexisting IgG1 PCs associated with clinical response are expanded after PD-1 blockade

We hypothesized that skewing of IgG1-producing PCs is linked to clinical response, given their overrepresentation in single-cell data from responders (Extended Data Fig. 1i). To test this, we used complementary bulk RNA-seq of paired pretreatment biopsies and posttreatment resected tumors from the discovery cohort. Principal component analysis showed that gene expression profiles were markedly different between responders and non-responders (Fig. 1f,g and Extended Data Fig. 1j–k).

In posttreatment tumors, *IGHG1* emerged as one of the top upregulated genes. Its expression was already elevated in pretreatment samples from responders and increased significantly after therapy, whereas it remained unchanged or decreased in non-responders (FDR < 0.01; Fig. 1h,i). Projection of the single-cell signatures onto bulk data emphasized the increase of PCs in responders (FDR < 0.01) (Fig. 1j). Thus, these results suggest that skewing toward an IgG1 signature may exist at baseline (pretreatment) in anti-PD-1 responders, which was significantly amplified after ICB treatment and was highly associated with response.

### ICB responders have clonally expanded IgG1 PCs trafficking between the tumor and draining lymph node

**Fig. 2:**
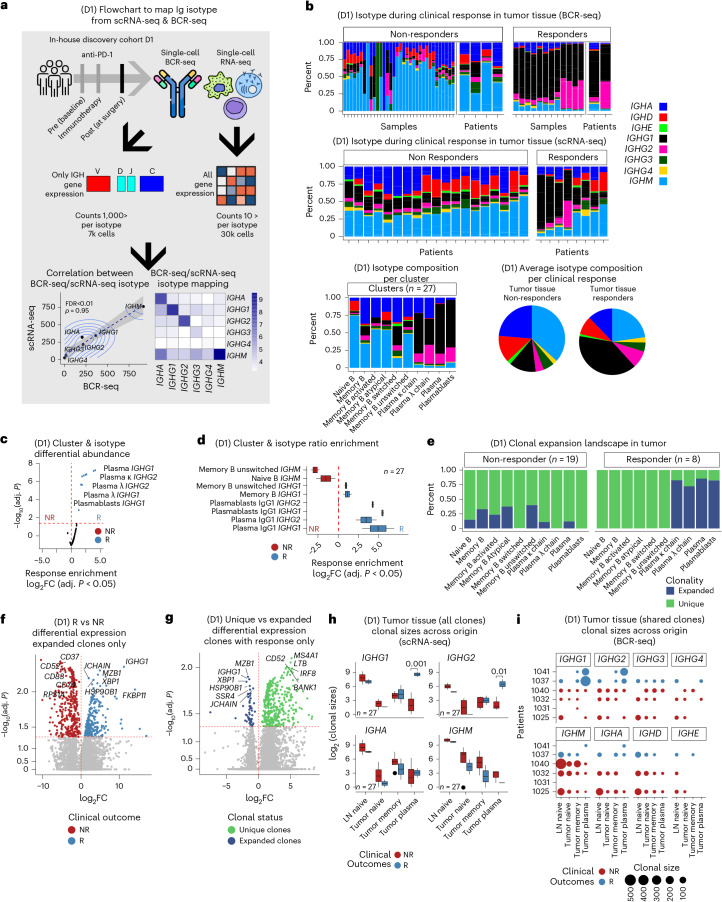
Clonality. **a**, Flowchart illustrating Ig isotype mapping for 37,000 single B cells, including 30,000 cells with paired BCR-seq and 7,000 additional cells mapped via gene-expression-based inference. Bottom panels show correlations between isotype assignments from gene expression and scBCR-seq for the 30,000 cells with paired data. **b**, Stacked bar plots showing isotype composition per sample and per patient. Multiple samples per patient were sequenced to ensure reproducibility. Single-cell BCR-seq data include six patients with HCC treated with ICB (two responders and four non-responders). Using scRNA-seq-based isotype inference, the analysis was expanded to 8 responders and 14 non-responders. The *y* axis represents the proportion of cells (0–1). Consistent across BCR-seq and scRNA-seq-rescued isotypes, PC clusters are enriched for IgG1/IgG2, whereas naive and memory B cell clusters predominantly express *IGHM* and *IGHA* (bottom bar plots and pie charts). **c**, Volcano plot showing Dirichlet regression comparing responder and non-responder frequencies across clusters and isotypes (from both scRNA-seq and scBCR-seq). The log-transformed fold changes and *P* values were obtained from log-likelihood tests; adjusted *P* values were computed using Benjamini−Hochberg correction. PCs and IgG1/IgG2 responses are significantly enriched in responders. **d**, Box plot (as defined in Fig. 1c) showing increased IgG1^+^ PC representation in tumor tissue of responders with available BCR-seq. Ratios were estimated using Dirichlet regression with log-likelihood testing. **e**, Stacked bar plot of expanded clones per cluster, defining expansion as two or more cells per clonotype. Expansion occurs specifically in PCs of responders. **f**, Differential expression (two-sided Wilcoxon test, Benjamini−Hochberg adjusted) comparing expanded clones in responders versus non-responders. *IGHG1*, *MZB1*, *JCHAIN* and *XBP1* are associated with clinical response. **g**, Differential expression comparing expanded versus non-expanded cells in responders shows expansion-associated upregulation of *IGHG1*, *MZB1*, *JCHAIN* and *XBP1*, whereas non-expanded cells upregulate *LTB*, *MS4A1*, *CD52* and *IRF8* (Benjamini−Hochberg adjusted). **h**, Box plots showing clonal sizes after filtering for shared CDR3 sequences within clonotypes and clusters across 27 patients. Clonal sizes increase for IgG1, IgG2, IgA and IgM in both B and plasma compartments (two-sided Wilcoxon test). **i**, Clonal sizes of expanded (≥2 cell) clones from bulk BCR-seq in two responders and non-responders show increased IgG1 expansion in responders. LN, lymph node.

**Extended Data Fig. 2:**
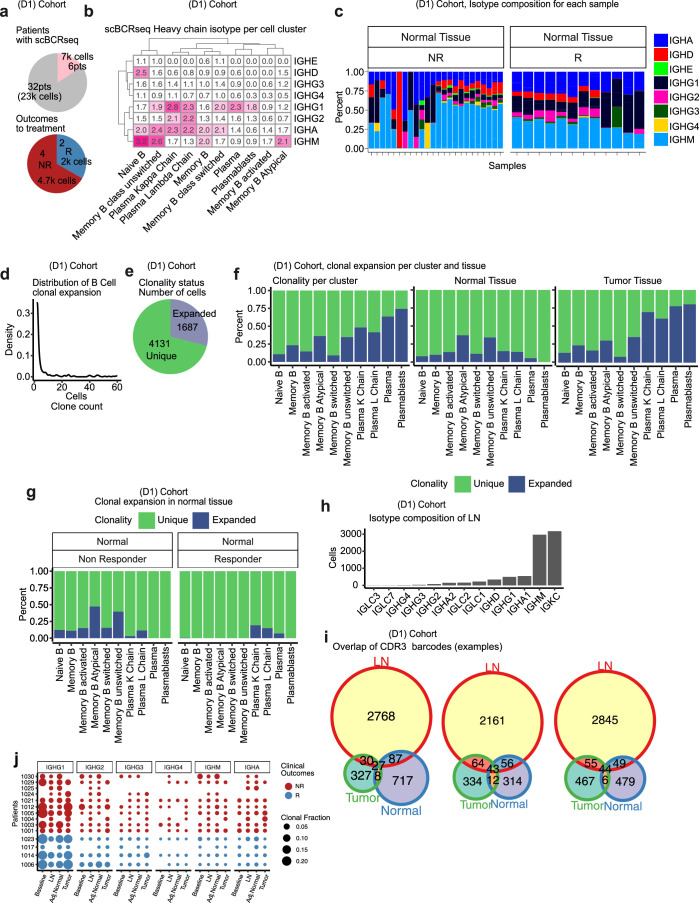
Complementary BCR analysis. **a**. Pie chart showing on top the number of patients and cells with available scBCRseq. Bottom shows the number of responder and non-responders. **b**. Heatmap showing the average log10 expression per isotype for every cluster of cells identified. **c**. Percent of plasma cell isotypes identified in responder and non-responders in normal tissue; differences did not reach statistical significance. The percent values are shown in the y-axis, while the isotype labels are shown by colors. **d**. Distribution of clonally expanded B and plasma cells. **e**. Pie chart showing the total number of cells classified as expanded or unique. **f**. Stacked bar plot panel showing expansion levels for all the data set, normal tissue and tumor issue, from left to right, respectively. **g**. Stacked bar plot showing the composition of expanded and non-expanded cells in normal tissue for responders and non-responders. **h**. Bar plot showing the isotype composition of cells in the lymph node. **i**. Overlap of CDR3 barcodes between LN, tumor and adjacent normal tissue samples for 3 patients, samples are matched per patient. **j**. Y-axis average shared clonotypes per individual identified in cells across different clusters (LN (lymph node), Naïve, Memory and Plasma compartments) for IgG1, IgG2, IgM and IgA. This figure shows that plasma IgG1 and IgG2 have larger clones in responders, while Naïve or Memory cells with IgM or IgA isotype are most commonly present in non-responders.

Using a combination of BCR-seq with scRNA-seq, we investigated clonal expansion and immunoglobulin isotype (Fig. 2a). We found that plasmablasts and PCs were largely of the *IgG1* and *IgG2* subclass (FDR < 0.01), whereas other memory and naive B cells were an admixture of *IgM*, *IgD*, *IgA* and, to a much lesser degree, the other *IgG* subclasses (Fig. 2b, top). When stratifying by ICB response, *IgG1* and *IgG2* PCs were almost exclusive to responders, whereas *IgM*, *IgA* and *IgD* dominated in non-responders (Fig. 2b, bottom, and Extended Data Fig. 2a,b). Irrespective of ICB response, 26% of total B cells and PCs were expanded (more than one cell per clone) (Extended Data Fig. 2d,e). Responders exhibited marked intratumoral IgG1^+^ PC and plasmablast expansion, unlike non-responders, who showed memory B cell expansion (Fig. 2c–e and Extended Data Fig. 2c,f,g). *IGHG1*, *MZB1*, *JCHAIN* and *XBP1* were among the top genes enriched in clonally expanded cells from responders (Fig. 2f). In responders, clonally expanded cells were enriched for IgG1^+^ PCs, whereas non-expanded cells expressed *MS4A1* (encoding CD20) (Fig. 2g). Together, these data suggest a tumor-specific clonal expansion of IgG1 antibody-producing cells in ICB responders.

Next, we investigated whether B cell differentiation and clonal expansion occur at the primary tumor site or in draining lymph nodes. In lymph nodes, IgM was the dominant isotype (Extended Data Fig. 2h). Although unique CDR3 sequence overlap among lymph node, tumor and adjacent liver samples was limited within individual patients (Extended Data Fig. 2i), clonal tracking revealed significantly larger IgG1^+^ and IgG2^+^ tumor PC clones in responders compared to non-responders (FDR < 0.01; Fig. 2h). The shared CDR3 clonotypes across cellular compartments (lymph node, naive, B memory and plasmablast/PC) revealed that clonally expanded cells with the same CDR3 sequence could be found at the lymph node as well as the tumor site (Fig. 2i and Supplementary Table 1), thus suggesting trafficking of these expanded clones between the tumor and the draining lymph node. Further inspection of the bulk sequencing data validated the expansion of *IGHG1* phenotype, including evidence of the same CDR3 barcodes before and after treatment in both responders and non-responders (Extended Data Fig. 2j).

### Tumor microenvironment spatial distribution reveals PC-driven immunity in responders and memory B cell dysfunction in poor outcomes

**Fig. 3:**
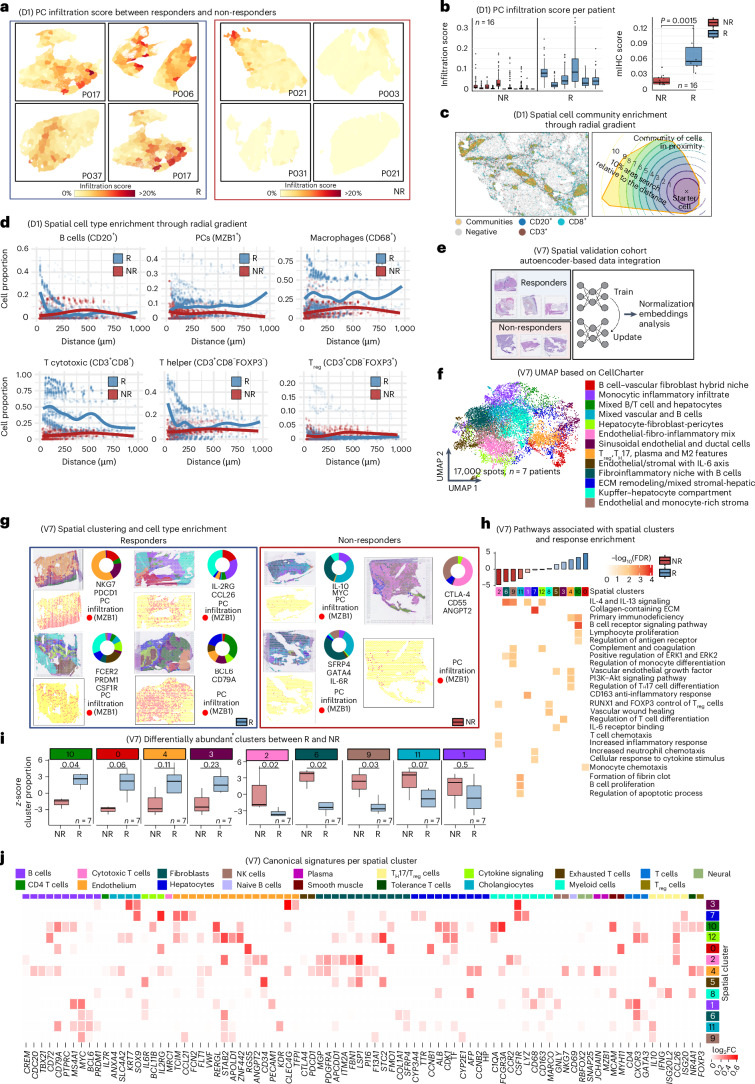
Spatial analysis of immune infiltrating cells. **a**, Representative examples showing increased PC infiltration in responders compared to non-responders, quantified as the percentage of PCs within unsupervised neighborhood regions derived from mIHC images. P, patient. **b**, Box plots (as defined in Fig. 1c) comparing PC infiltration scores between responders (*n* = 6) and non-responders (*n* = 10), shown both per patient and as averaged scores across regions (*P* < 0.05, two-sided Wilcoxon rank test). The left plot displays individual regions per patient; the right plot summarizes patient-level averages across 16 total patients. **c**, Unsupervised identification of immune cell aggregates from mIHC using a spatial enrichment analysis. A radial gradient approach quantifies local immune communities by evaluating up to three markers within a 10-µm distance (approximately one cell diameter) from a reference cell. Community size is estimated by the area captured within the radial gradient. **d**, Spatial enrichment of immune populations within aggregates in responders versus non-responders. Responders show increased PCs (MZB1^+^), cytotoxic T cells (CD3^+^CD8^+^) and macrophages (CD68^+^), whereas non-responders show higher levels of B cells (CD20^+^) and regulatory T (T_reg_) cells (CD3^+^CD8^−^FOXP3^+^). **e**, Schematic of spatial transcriptomics integration using an autoencoder-based framework to cluster spatial spots. **f**, Spot clustering results identifying 13 spatial clusters across approximately 17,000 spots from seven patients (four responders and three non-responders). **g**, Enrichment of responder-associated clusters and top markers per most abundant cluster for each patient, demonstrating sample-level concordance between spatial clusters and biological phenotypes. **h**, Top pathways associated with each spatial cluster, highlighting cellular, molecular and functional programs tied to distinct microenvironmental niches. **i**, Box plots (as in Fig. 1c) showing cluster-level enrichment patterns stratified by clinical response. Responders exhibit higher representation of plasma, T cell and myeloid-associated clusters, whereas non-responders are enriched for regulatory and dysfunctional phenotypes. Statistical significance was assessed with a two-sided Wilcoxon rank test across seven patients (four responders and three non-responders). **j**, Canonical cell markers enriched per cluster, confirming the identity of dominant immune and stromal populations defining each spatial niche. ECM, extracellular matrix.

Using multiplex immunohistochemistry (mIHC) and computational tools^19^, we examined the spatial distribution of the B cells (CD20^+^), PCs (MZB1^+^) and other immune cells in the tumor and adjacent liver in 17 mIHC biopsies (6 responders and 11 non-responders) from our discovery cohort. We observed that among anti-PD-1 responders, the MZB1^+^ PCs were highly infiltrative throughout the tumor parenchyma compared to the PCs in non-responders (Fig. 3a,b). Next, we used a radial binning approach to define cell communities or immune aggregates in an unsupervised fashion (Fig. 3c). Here, responders showed enrichment of CD3^+^CD8^+^ T cells, CD68^+^ macrophages and MZB1^+^ PCs. Conversely, CD20^+^ B cells were found within the lymphoid aggregates or within the stromal compartment of the tumor rather than admixed with tumor parenchyma, reinforcing PC expansion as a hallmark of effective ICB response (Fig. 3d).

Next, we applied an single-cell variational inference-based autoencoder to integrate the spatial transcriptome cohort^3^ and then used CellCharter to identify spatial clusters constituted by multiple cell types (Fig. 3e,f). In ICB responders, we saw a marked enrichment of IgG1^+^ PCs confined to an immune-rich T cell/B cell/vascular fibroblast hybrid niche, with significant upregulation of IL-4 and IL-13 signaling (Fig. 3g,h). Pathway analysis of these clusters revealed a pro-BCR/TCR signaling hub, marked by *MZB1*, *IL2RG*, *FCER2*, *PRDM1* and *CD79A*, consistent with a highly immunogenic, PC-driven microenvironment (Fig. 3h,i). By contrast, non-responders harbored focal accumulations of CD27^+^ memory and dysfunctional B cells within fibroinflammatory stromal regions. These niches were accompanied by regulatory T, T helper 17 (T_H_17) and monocyte signatures, collectively defining an immunosuppressive ‘stromal memory/exhausted B cell reservoir’ (Fig. 3g–j).

### IgG1 PCs in tumor and lymph nodes increase after radiation therapy and PD-1 checkpoint blockade

**Extended Data Fig. 3:**
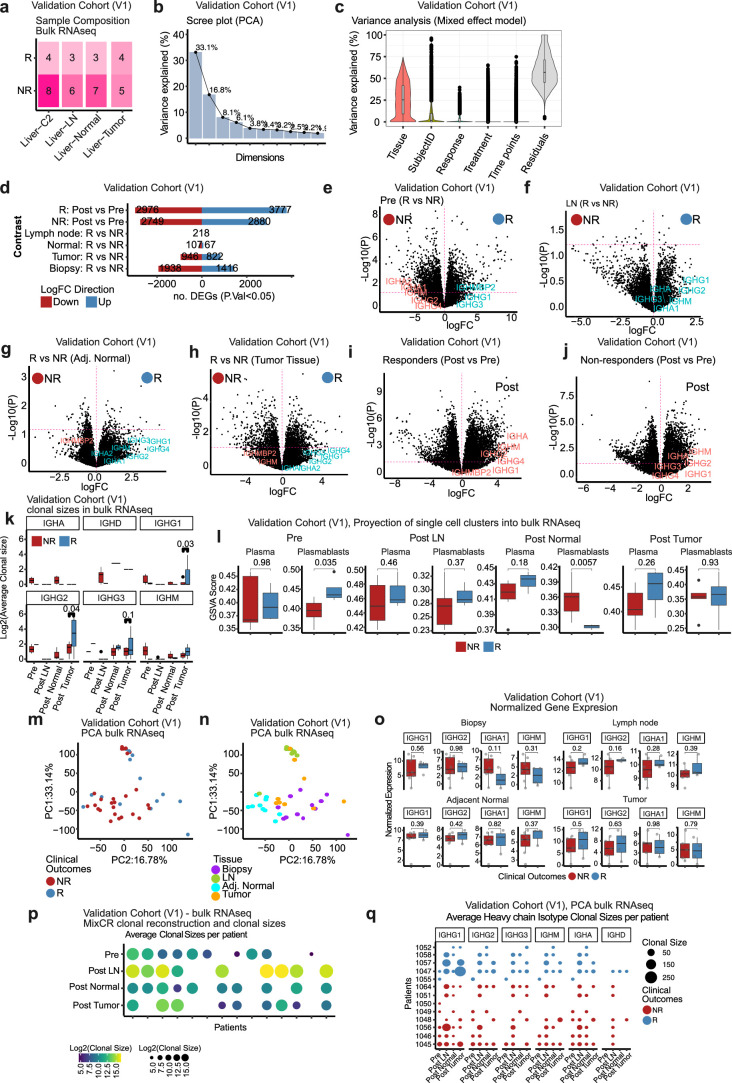
Complementary bulk RNA-seq analysis. Bulk RNAseq (Radiation+PD-1 cohort) **a**. Table showing the number of samples per tissue type and response. **b**. Principal component analysis showing the percent variance explained by every principal component. **c**. Violin plot from the variance analysis showing the effect of covariates modeled in this analysis. Tissue type was the largest source of variance for this bulk RNAseq. **d**. Barplot showing the number of differentially expressed associated with either response or non-response per comparison using moderated t test statistics. **e**–**j**. Volcano plots showing the direction and position of IG heavy chains in the differential expression gene (DEG) results. In tissue comparisons, there was no difference between responders and non-responders for *IGH* isotypes. However, changes over time for either responders or non-responders showed an increase of *IGH* expression (i-j). The p-values were calculated using a two-sided moderate t test. **k**. Boxplots (same definition as 1c) showing the average clonal sizes for responders and non-responders. Median increase in *IGHG1* and IGHG2 reached nominal statistical significance (pval<0.05), but not FDR correction when using a two-sided Wilcoxon rank test. **l**. Boxplots (same definition as 1c) showing the results from a projection of previously identified single cell signatures for Plasma and Plasmablast clusters into bulk RNAseq showed a significant increase when using a two-sided Wilcoxon rank test at the time of biopsy and borderline significant changes post treatment for plasma cells. Single cell RNAseq (Radiation+PD-1 cohort). **m**, **n**. Principal component analysis showing PC1 and 2 reflecting approximately 50% of the variance distinguishing clinical response and tissue type. **o**. Box plots (same definition as 1c) showing the normalized expression levels of IGHX heavy chain genes corresponding to either responder and non-responder. The p-values were estimated using a two-sided Wilcoxon rank test **p**. Dotplots showing the average clonal sizes per patient for the different tissue types and timepoints available. **q**. Dotplots showing the heavy chain clonal sizes per isotype that are shared between tissue origin and clinical response status.

To validate these findings in an independent cohort^18^, we analyzed B cells and PCs from patients with HCC treated with neoadjuvant stereotactic radiation followed by anti-PD-1 therapy (biopsy, lymph node, tumor and adjacent normal) (Fig. 1a and Extended Data Fig. 3a–c). In bulk sequencing, differential gene expression between responders and non-responders was most pronounced in pretreatment biopsies and posttreatment tumors but minimal in lymph nodes and adjacent normal tissues (Extended Data Fig. 3d–h). After treatment, total immunoglobulin increased, with responders showing selective IgG1 enrichment (Extended Data Fig. 3i,j). The bulk data also showed an increase in *IGHG1*, *IGHG2* and *IGHG3* expression in tumor tissue of responders (Extended Data Fig. 3k). As expected, we observed higher plasmablast and PC levels in responder biopsies (FDR < 0.2) (Extended Data Fig. 3l–n). Interestingly, the increase in *IGHG1*−*IGHG4* and decrease in *IGHM* were also observed in responders across tissues (FDR < 0.2) (Extended Data Fig. 3o). Clonal size analysis showed that the largest clones were identified in lymph nodes, followed by pretreatment and posttreatment tumor tissue (Extended Data Fig. 3p). Shared clonotypes were observed between all three posttreatment tissue types and pretreatment biopsies (Extended Data Fig. 3q).

**Fig. 4:**
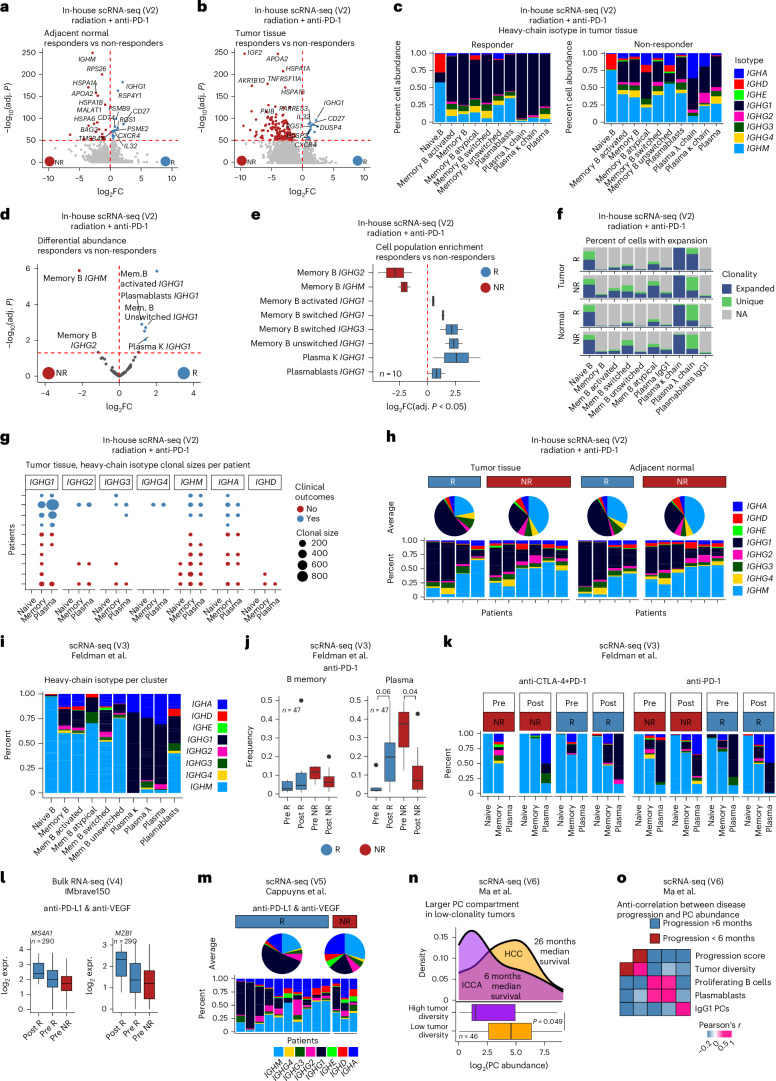
Validation sets. V2 cohort (radiation + anti-PD-1): **a**,**b**, Differential expression analysis of adjacent normal and tumor tissues revealed *IGHG1* as the most significant gene associated with response, using a two-sided moderated *t*-test. **c**, Tumor heavy-chain isotype composition by cell cluster showed *IGHG1* enrichment in responders. **d**, Differential abundance modeling using Dirichlet regression and log-likelihood testing identified *IGHG1*^+^ PC and B cell phenotypes as significantly enriched in responders. **e**, Box plots (as in Fig. 1c) illustrate the magnitude and heterogeneity of cluster enrichment in responders versus non-responders across 10 patients (four responders and six non-responders), with significant findings at FDR < 0.05 (Benjamini−Hochberg correction). **f**, Proportions of clonally expanded cells (BCR-seq derived) showed that most cells were expanded in both groups, with no significant difference in overall expansion prevalence. **g**, Dot plots of shared heavy-chain clonal sizes per patient demonstrated larger *IGHG1* clonal expansions in responders. **h**, Responders showed dominant and expanded IgG1 PC isotypes in both tumor and adjacent normal tissue. V3 cohort (anti-PD-1 and CTLA-4 plus PD-1): **i**, Heavy-chain isotype analysis confirmed that PC isotypes were predominantly IgG1. **j**, Box plots comparing pretreatment and posttreatment samples showed that responders increase PC abundance over time, whereas non-responders decrease; memory B cell frequencies remained unchanged (Wilcoxon test). **k**, Both PD-1 alone and CTLA-4^+^ PD-1 therapies induced a responder-specific plasma IgG1 signature. V4 cohort (IMbrave150): **l**, Bulk RNA-seq showed nominal posttreatment increases in CD20 (*MS4A1*) and *MZB1* expression in responders although not statistically significant due to expression heterogeneity (moderated *t*-test). V5 cohort (anti-PD-1 and anti-VEGF-A): **m**, Responders receiving VEGF-A blockade in combination with PD-1 therapy also exhibited increased plasma IgG1 abundance relative to non-responders. V6 cohort (multiple treatments in HCC and ICCA): **n**, Box plots show that HCC samples contain higher PC abundance than ICCA samples and display slower progression times (Wilcoxon test). **o**, Across samples, disease progression positively correlated with tumor diversity scores and inversely correlated with PC abundance.NA, not available.

**Extended Data Fig. 4:**
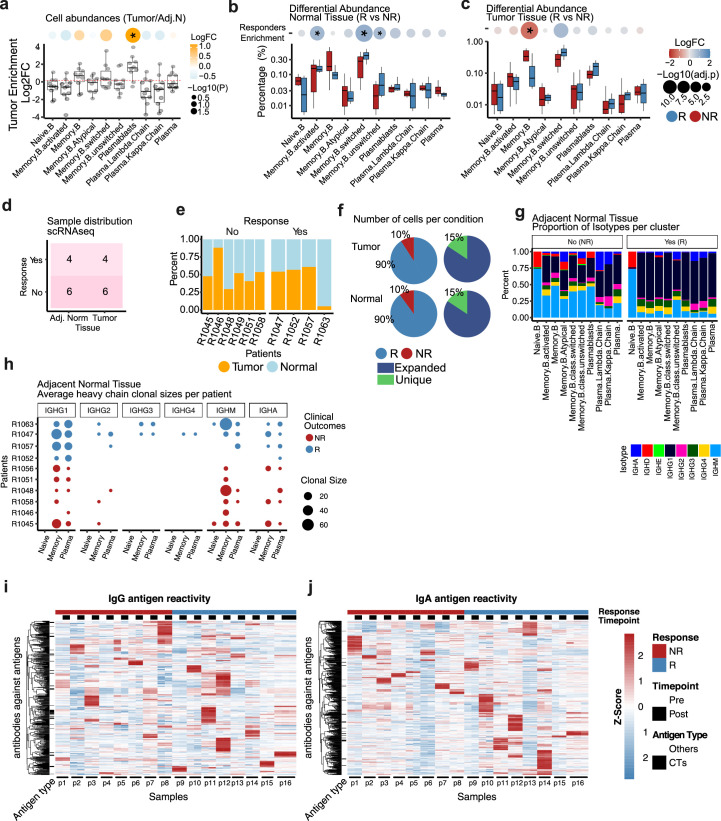
Complementary single-cell and seromics analysis in validation cohort. Validation cohort, Radiation+PD-1 treatment. **a**. Tumor enrichment in normal and adjacent tumor tissue using Dirichlet regression. The number of samples analyzed was 20. The stars and circles above the figure represent the log2FC and pvalue calculated using a log-likelihood test. **b**, **c**. Boxplot (same definition as 1c) showing the Dirichlet regression and log-likelihood test comparing responders and non-responders in either normal or tumor tissue for 8 responders and 12 non-responders. Single cell RNAseq results. **d**. Sample distribution in validation cohort scRNAseq data. **e**, **f**. Principal components of the RNAseq profiles labeled for response and type of treatment, respectively. Both figures capture the variance between samples represented as distances between each point. **c**. Normalized gene expression for heavy chains *IGHG1-2*, A and M for biopsies (pre-treatment), and post-treatment lymph nodes, adjacent normal and tumor tissues. **g**. Dotplot showing the average clonal sizes between tissues for every patient regardless of response and IG heavy chain isotype. **h**. Dotplots showing the heavy chain clonal sizes that are shared between tissues for every IG isotypes stratified per response. **i**, **j**. IgG antibody reactivity against human antigens including CTs, tumor antigens and others. Top bar shows response in red (NR) and blue (R), while lateral bar shows CTs in color black. The scale is the normalized log2 titers converted to z-score across samples for easier visualization.

Consistent with our findings, validation cohort scRNA-seq revealed significantly enriched plasmablasts in tumor tissue (FDR < 0.05) (Extended Data Fig. 4a). Activated B memory, class-switched and unswitched cells were also enriched in responders’ adjacent normal tissues (FDR < 0.05) (Extended Data Fig. 4b). In tumor tissue of responders, there was a minor increase in PCs (FDR < 0.2), whereas, in non-responders, there was a clear increase in B memory cells (FDR < 0.01) (Extended Data Fig. 4c). However, differential expression analysis recapitulated *IGHG1* as the top marker of clinical response in both tumor and adjacent liver (Fig. 4a,b). Contrary to our discovery cohort, this independent validation cohort showed similar B cell and PC numbers between adjacent normal and tumor tissue (Extended Data Fig. 4e). Notably, most of the sequenced cells were already clonally expanded (Extended Data Fig. 4f–h). However, once again, the IgG1 isotype dominated across all PC and non-naive B cell phenotypes in tumor tissues of responders, whereas non-responder B cells and PCs were more prevalent for IgM and IgA (Fig. 4c). Regardless of tissue, differential abundance analysis showed that *IGHG1* isotype, activated memory B cells, PCs and plasmablasts were associated with response, whereas IgM and IgG2 in memory B cells were enriched in non-responders (Fig. 4d,e). Although clonal expansion sizes were similar between B cells and PCs, tracking shared CDR3 clones revealed greater memory-to-plasma compartment transitions in responders, with exceptionally large *IGHG1* clones (Fig. 4f,g). Together, these results show that IgG1 PCs account for more than 50% of total isotypes identified in responders and for less than 30% in non-responders (Fig. 4h).

### IgG1 skewing is associated with response in other ICB-responsive tumors

**Extended Data Fig. 5:**
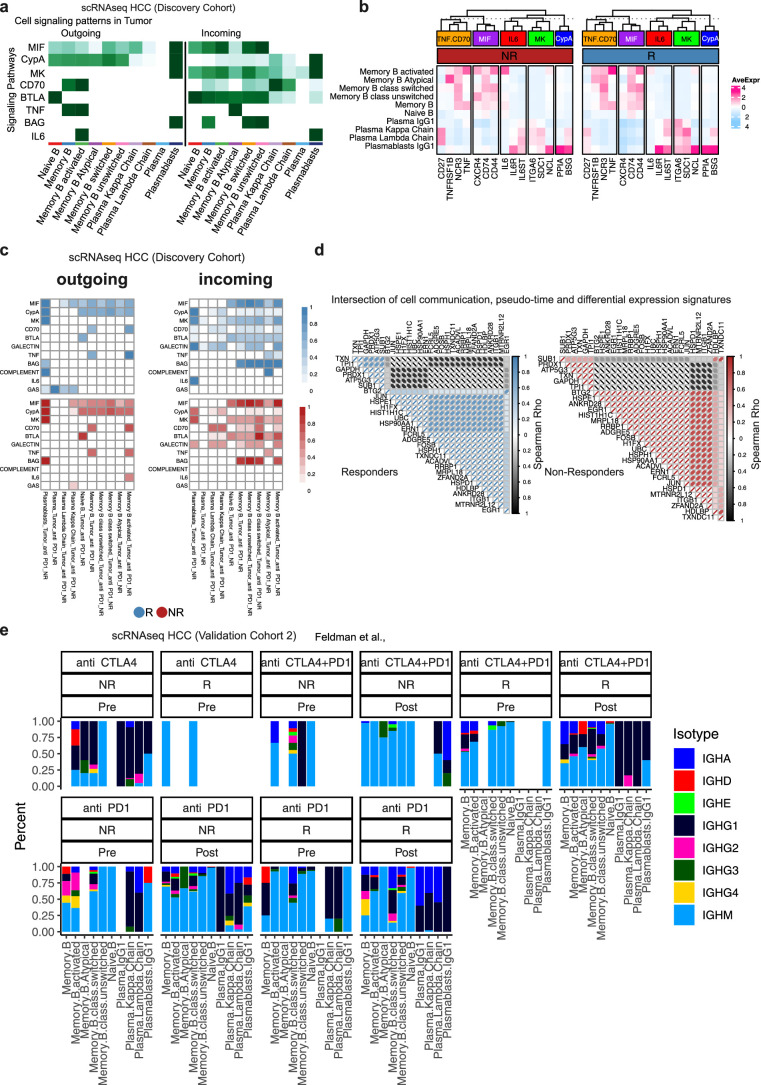
Complementary cell–cell interaction analysis. **a**. Heatmap showing incoming and outgoing signaling pathways between B or plasma cell clusters in tumor tissue calculated using the algorithm cellchat. **b**. Heatmap showing the average expression per category of cells and specific genes corresponding to signaling pathways with FDR < 0.01. **c**. Heatmap showing incoming and outgoing signaling pathways between B or plasma cell clusters in responders and non-responders calculated using the algorithm cellchat. **d**. Spearman Correlation plots showing the directionality and strength of the association between differentially expressed genes that are also high in Moran I’s score for responders and non-responders (FDR < 0.05 & Moran I > 0.5). **e**. Barplot showing the isotype composition of immunoglobulin heavy chain stratified by responders, treatment and pre/post tissues in Feldman et al. re-analysis.

To further extend our observations, we analyzed public datasets and found that patients with melanoma who responded to PD-1 with or without CTLA-4 blockade^3^ also showed expansion of PCs after treatment and a decrease in non-responders, despite a larger non-responder PC compartment before treatment, potentially due to low number of cells in this study (Fig. 4i–k). Furthermore, inspection of the isotype also showed IgG1 enrichment in PCs of responders (Fig. 4i–k and Extended Data Fig. 5e). Next, we validated our findings in advanced HCC treated with anti-PD-1 and anti-VEGF-A therapies in two independent cohorts of unresectable HCC: IMbrave150 (ref. ^20^) and Cappuyns et al.^21^ (Fig. 4l,m). In both datasets, responders exhibited a skewing toward the IgG1 isotype, with IgG1 emerging as the dominant subclass. Another independent cohort of patients with HCC and intrahepatic cholangiocarcinoma (ICCA)^22^ showed that HCC tumors had a low tumor diversity score, linked to favorable outcomes, whereas ICCA tumors exhibited a high tumor diversity score, linked to a more aggressive phenotype and worse outcomes (6-month survival for ICCA versus 26-month survival for HCC). Notably, HCC samples had greater PC abundance, consistent with better progression-free survival in HCC than in ICCA (Fig. 4n,o). Together, these findings suggest that IgG1 PCs are strongly linked to ICB response.

### ICB responders produce IgG antibodies against cancer antigens

**Fig. 5:**
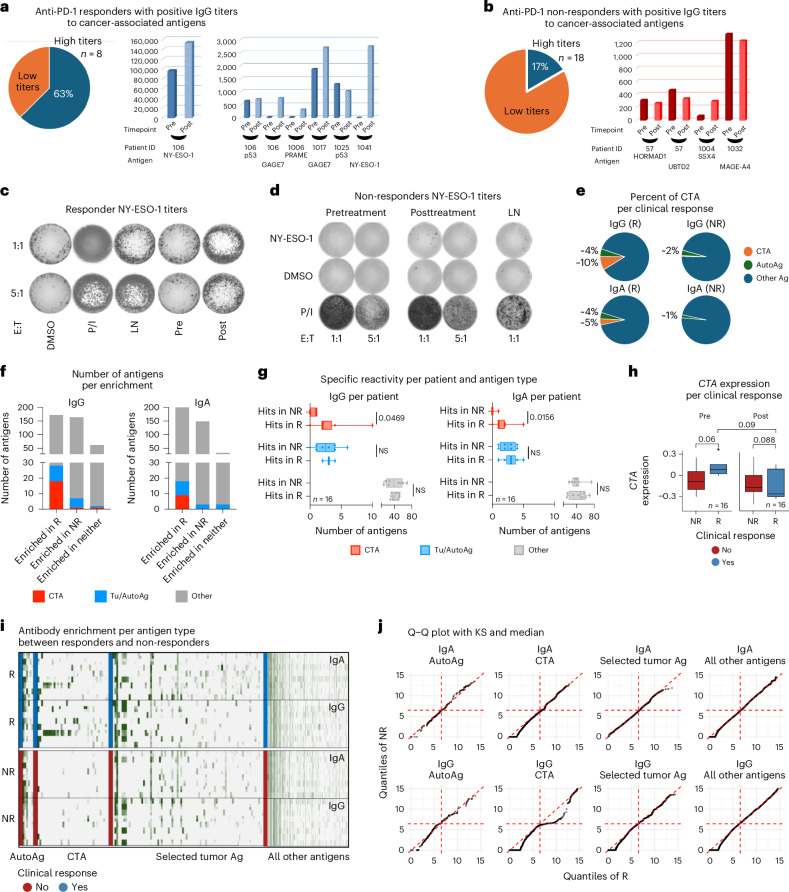
Cancer antigens and serological markers. **a**,**b**, Pie chart and bar plots showing that antibodies against cancer-associated antigens are predominantly enriched in responders compared to non-responders, respectively. **c**,**d**, ELISpot analysis of IFNγ-secreting cells in response to varying effector-to-target (E:T) ratios. The left panel shows wells with decreasing numbers of spots from top to bottom, corresponding to different ratios (1:1 and 5:1) for two conditions, indicating the frequency of cytokine-producing cells. The right panels represent different experimental conditions or treatments, with each row representing different replicates or conditions. Darker and more numerous spots indicate higher frequencies of cells secreting IFNγ. **e**, Similarly, a broader characterization using seromics indicates increased abundance of autoantibodies against CTAs in responders compared to non-responders. **f**, Number of antigens enriched between responders and non-responders. **g**, Analysis of 16 patients (8 responders and 8 non-responders) showed that an enrichment of antibodies against CTA, tumor-associated antigen (Tu/AutoAg) and other antigens was also higher in responders. Specifically, comparisons of CTA-specific IgG and IgA levels between responders and non-responders showed statistically significant differences (*P* = 0.04 and *P* = 0.01, respectively), as determined by the Wilcoxon rank-sum test and visualized by box plots (same definition as in Fig. 1c). **h**, Box plots (same definition as in Fig. 1c) showing the *CTA* gene expression signature divided by timepoint (pre or post) and clinical response (responders and non-responders); paired *t*-test and Wilcoxon rank test were used to estimate the significance between both groups; only paired *t*-test results are shown in the figure. **i**,**j**, The increase in autoantibodies against CTAs in responders, specifically in the IgG1 and IgA isotypes, was assessed using a heatmap to visualize relative abundance patterns across samples, violin plots to display the distribution and variability of antibody levels and quantile−quantile (Q−Q) plots to evaluate deviations from normality and highlight differences in distribution between responders and non-responders. KS, Kolmogorov–Smirnov test; NS, not significant; P/I, phorbol myristate acetate (PMA) and ionomycin positive control.

Given the enrichment of IgG1 PCs in ICB responders, we explored whether these patients potentially generated antitumor antibodies detectable in the patients’ serum. Thus, we investigated serum samples collected before and during treatment from our discovery cohort, testing against a panel of 20 tumor-associated antigens. This panel included common CTAs, mutational antigens and stem-cell-associated antigens. A higher proportion of responders (63%) exhibited antitumor IgG antibodies in their serum compared to non-responders (17%), with the IgG antibodies primarily belonging to the IgG1 subclass and targeting CTAs such as MAGE-A, GAGE7, PRAME and NY-ESO-1 (Fig. 5a,b). These data suggest that responders with circulating IgG antibodies against CTAs generally had higher titers than non-responders. Conversely, IgG titers in non-responders were less dynamic, showing minimal changes after anti-PD-1 treatment. IgG1 antibodies targeting CTAs can enhance antigen presentation by antigen-presenting cells and potentially prime CD8^+^ T cells through immune complexes and cross-presentation^23,24^.

Next, to investigate T cell responses, we performed an ELISpot assay with CD8^+^ T cells isolated from pretreatment and on-treatment peripheral blood mononuclear cells (PBMCs), resected tumors and draining lymph nodes with detectable NY-ESO-1 antibodies. The responder’s CD8^+^ T cells showed significant IFNγ, whereas the CD8^+^ T cells from the non-responder, who had predominantly IgA, showed only minimal reactivity (Fig. 5c,d). Notably, the responder had circulating NY-ESO-1 antibodies before ICB treatment, but IFNγ production by CD8^+^ T cells was observed only in on-treatment samples, suggesting that antitumor B cell response may precede T cell response, as in previous reports^23,24^.

To assess if serum autoantibodies target cancer antigens more than other autoimmune targets, we performed seromic profiling (IgG and IgA against approximately 20,000 antigens, including 186 CTAs; Supplementary Table 1) on 32 paired pretreatment and posttreatment samples from a discovery HCC neoadjuvant PD-1 cohort (8 responders and 8 non-responders). We observed that IgG autoantibodies and, to a lesser extent, IgA were enriched for CTAs in responders compared to non-responders (Fig. 5e).

Surprisingly, reactivity to CTA was enriched for response, as antibodies detected to another approximately 400 other known non-CTA tumor antigens (including p53) had similar prevalence in responders and non-responders for IgG and IgA (Fig. 5f). Notably, almost all antigen-specific antibodies were unique to individual patients and were found before treatment and after treatment, although some increases in reactivity were noted after treatment (Extended Data Fig. 4i,j). Looking in individual samples, only CTA-specific antibodies showed a significant increase with clinical benefit in total number of reactivities (on average, 2−3 hits in responders versus 0−1 hits in non-responders) (Fig. 5g). In parallel, we did not observe correlation between gene expression of CTAs and autoantibodies, suggesting that immunogenicity is more important than expression alone (Fig. 5h). Finally, the increase in autoantibodies against CTAs in responders (*P* < 0.05) in IgG was identified as specific for CTAs compared to IgA, autoantigens and other antigens (Fig. 5i,j). Together, these results support the notion that antibody production and reactivity against CTAs are indicators of clinical response to ICB, in parallel to an increase of IgG1 PCs.

### Plasma IgG1 signature associated with improved survival in immunotherapy

**Fig. 6:**
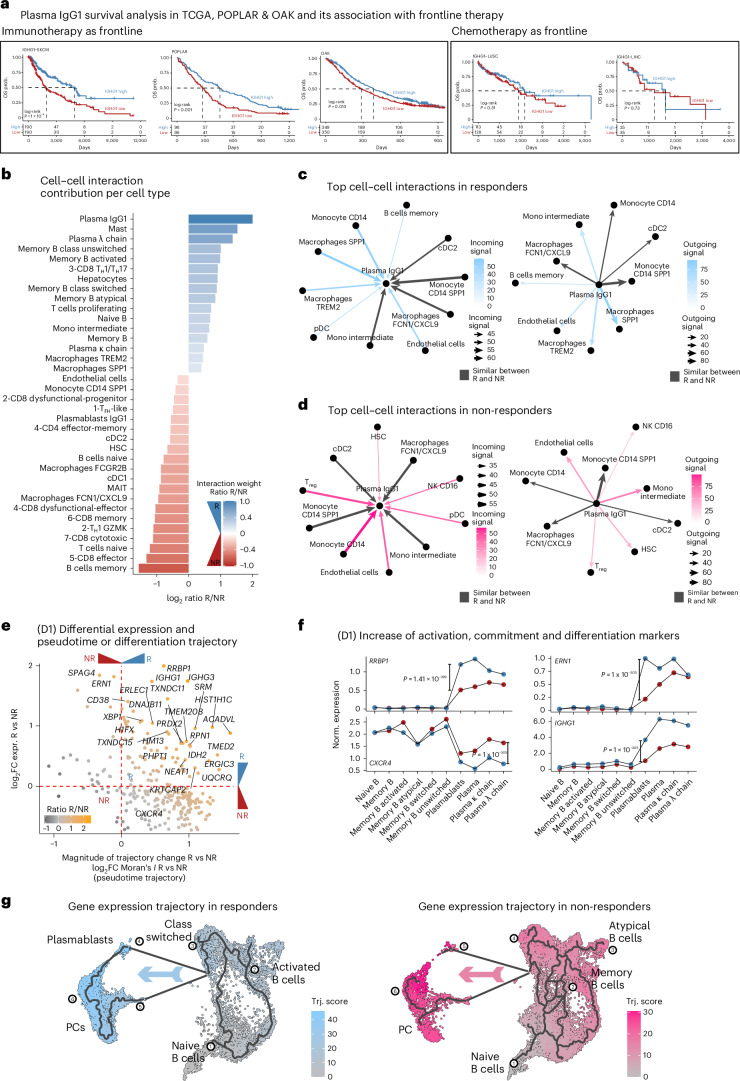
Survival analysis and potential cell−cell interactions and mechanisms behind IgG1 phenotype. **a**, Kaplan−Meier survival analysis from TCGA and clinical trial cohorts (POPLAR and OAK) stratified by high versus low IgG1 expression levels across multiple cancer types (SKCM, LUSC and LIHC). High IgG1 expression is associated with improved survival in several contexts (log-rank *P* values shown). OS, overall survival. **b**, Cell−cell interaction contribution scores for each cell type, highlighting plasma IgG1 cells as major contributors in responders. Bar plot shows interaction weight ratios (R/NR) per interaction pathway, color coded by interaction strength. cDC1/2, conventional type 1/2 dendritic cells; HSC, hematopoietic stem cells; MAIT, mucosal-associated invariant T cells; T_F__H_, follicular helper T cells. **c**,**d**, Top cell−cell interactions enriched in responders (blue) and non-responders (red), based on ligand−receptor analysis. Arrows indicate the directionality and magnitude of cell type interactions, with plasma IgG1 cells and myeloid compartments prominently engaged in responders. **e**, Trajectory analysis and differential expression overlap between responders and non-responders showing genes significantly changing between compartments and between responders and non-responders (FDR < 0.05 and Moran’s *I* > 0.5, estimated using two-sided Wilcoxon rank test and the Monocle 3 pseudotime package). **f**, Line plots showing the pseudobulk normalized median expression of *RRBP1*, *CXCR4*, *ERN1* and *IGHG1* along the trajectory path traced using the Monocle 3 algorithm and Moran’s *I* statistical tests (FDR < 0.05 and Moran’s *I* > 0.5 were estimated in **e** using two-sided Wilcoxon rank test and Monocle 3 pseudotime package). **g**, RNA trajectories build using either responders (blue) or non-responders (red) showing that responders differentiate mostly in activated and switched B cells leading to plasmablasts and PCs, whereas non-responders have higher pseudotime scores in atypical and memory B cells. Trj., trajectory.

To explore the relevance of IgG1 PC expression in patient survival, we used independent immunotherapy clinical trials (approximately 1,500 patients)^25–27^. Notably, high *IGHG1* expression was associated with improved overall survival in multiple datasets, including skin cutaneous melanoma (SKCM) (TCGA)^25^ and POPLAR^26^ and OAK^26^ trials (patients with NSCLC treated with anti-PD-L1) (Fig. 6a). Notably, non-immunotherapy trials showed no effect of *IGHG1* on survival (lung squamous cell carcinoma (LUSC) and liver hepatocellular carcinoma (LIHC)). These results indicate that chemotherapy-treated cancers have no clear link between clinical response and *IGHG1*.

### B cells differentiate toward plasma IgG1 in clinical responders

Finally, we evaluated the immune cell contributions in cell−cell communication networks. IgG1 PCs, alongside specific macrophage and T cell subsets, were key drivers of interaction strength in responders (Fig. 6b). Responders showed stronger interaction scores among IgG1 PCs, plasmacytoid dendritic cells and macrophages, whereas non-responders had enriched interactions involving monocytes, regulatory T cells, NK cells and immature dendritic cells (Fig. 6c,d). These findings suggest IgG1-skewed PCs foster a favorable immunogenic microenvironment through enhanced immunostimulatory signaling, potentially improving immunotherapy outcomes. We also identified key pathways potentially driving plasma IgG1 differentiation, including IL-6, TNF, MK, CD70, BTLA, MIF, BAG and CypA, which were enriched as both incoming and outgoing signals (FDR < 0.05) (Extended Data Fig. 5a–d and Supplementary Table 2).

By integrating these signaling results with differential gene expression and pseudotime analysis, we identified genes strongly associated with clinical outcomes. Responders showed higher expression of genes related to PC differentiation, such as *ERN1* and *RRBP1*, whereas non-responders showed increased *CXCR4* (Fig. 6e,f and Supplementary Table 2). These patterns suggest that ICB induces B cell activation and class switching, leading to plasmablast expansion and differentiation into IgG1-secreting PCs (Fig. 6f,g). By contrast, non-responders accumulate fewer diverse memory cells, atypical B cells and non-IgG1 PCs. In summary, we identified critical gene programs that support sustained B cell activation and plasma IgG1 differentiation, both of which are associated with favorable clinical response.

## Discussion

Although ICB is a cornerstone of cancer therapy, its effect on humoral immunity is not well understood. The presence of intratumoral B cells is strongly associated with positive ICB responses across various cancers^14^, but the specificity of these cells to tumor antigens and the mechanisms driving this benefit remain unclear^28,29^. We found that clinical responders were enriched in IgG1 PCs, which have high antitumor potential, similar to findings in colorectal cancer^30^. We observed a dynamic, tumor-specific differentiation of these IgG1 PCs, which were present at baseline and expanded upon ICB treatment. Conversely, non-responders had an abundance of naive and memory B cells, phenotypes that can contribute to cancer progression^31–33^. These data suggest that different B cell phenotypes are recruited to the tumor or lymph nodes^12,34^, and improper differentiation can worsen outcomes^35–38^. This is supported by observations that antigen-specific B cells differentiating into PCs are associated with improved ICB outcomes^39–41^.

The presence and expansion of circulating IgG1 antibodies against CTAs in responders further suggests a dynamic, antigen-driven adaptive immune response. We hypothesize that these antibodies enhance T cell induction through cross-priming^23,24^, which aligns with previous findings where CTA seropositivity correlates with ICB benefit^15–17,42,43^ despite being linked to worse prognosis^44–46^. Although both responders and non-responders have preexisting tumor-specific clonotypes, only responders show significant clonal expansion with immunotherapy. Correspondingly, PCs in responders heavily infiltrate the tumor parenchyma, mimicking CD8^+^ T cells, whereas infiltration in non-responders is sparse. These findings suggest that PCs in responders have active effector functions and that CTA-targeted therapies, such as vaccines combined with ICB, could be a promising strategy to stimulate these beneficial IgG1 responses^47^. Combined, these findings suggest that PCs of responders have active effector function compared to other subsets of B cells.

The B cells and PCs present in the tumor or adjacent tissues in non-responders had a diverse immunoglobulin repertoire, including IgA, IgG1−IgG4 and IgM without specific isotype enrichment, whereas, on the contrary, responders had an enrichment of IgG1 PCs and plasmablasts. Interestingly, studies have associated IgA plasmablasts derived from reprogramming by cancer-associated fibroblasts to be linked with poor clinical outcomes^48^. However, we noted that, in responders, not only was there a skewing toward IgG1 at baseline, but there was also an amplification of preexisting matching *CDR3* IgG1 cells after immunotherapy compared to their abundance in biopsies taken prior to treatment. The expansion of plasma IgG1 effector cells is potentially associated with immunotherapy induced type I interferon responses^49^. Together, these findings suggest that an IgG1 PC signature may serve as an important pretreatment biomarker. Moreover, the observed increase in IgG1 abundance after ICB indicates that this dynamic change is strongly associated with a favorable response to therapy. Incorporating these insights into current treatment strategies could involve promoting B cell differentiation toward IgG1-producing PCs by targeting pathways identified in this study, such as *IL6*, *TNF*, *RRBP1*, *ERN1* and *CD70* signaling. Therapeutic agents such as IL-6 agonists or TNF pathway activators warrant exploration as potential means to enhance IgG1 skewing, thereby potentially improving the efficacy of ICB.

A potential mechanism that favors the survival and expansion of plasma IgG1 involves the CyPA and Midkine signaling pathways, which help inhibit IL-6 degradation and promote survival through CD74, respectively^50,51^. In combination with proinflammatory conditions due to stimulation from myeloid cells through MIF, GAS, CXCL and Complement, IgG1-secreting PCs may be sustained during antitumoral response^52,53^. Elegant in vitro studies congruent with our trajectory analysis have emphasized the importance of sustained signaling of PCs and stimulation of ERN1 to achieve clinical response^54,55^. We observed significant clonal expansion after combination of radiation and ICB, suggesting that Ig-secreting PCs survived the radiation treatment, which was also reported to be associated with activation of *ERN1* and *XBP1* genes^56^.

These findings from patients with liver cancer treated with neoadjuvant ICB mirror observations in patients with advanced melanoma or NSCLC^3^ treated with PD-1 with or without CTLA-4 inhibitors from TGCA-SKCM^25^, POPLAR^26^ and OAK^27^ cohorts (immunotherapy as frontline). By contrast, cancers where chemotherapy is frontline are not associated with the IgG1 PC phenotype (TCGA-LUSC and TCGA-LIHC). The association between IgG1 skewing and improved survival across different tumor types underscores the broader implications of our results in guiding immunotherapy strategies^34,57–59^. The dominance of IgG1 subclass antibodies and their correlation with T cell activation highlights the interplay between humoral and cellular immune responses in mediating antitumor immunity. Overall, our study provides insights into the key role of B cell and PC responses in antitumor immunity and in response to ICB therapy, offering potential biomarkers for treatment stratification, and supports the hypothesis that tumor-specific humoral immunity is involved in ICB response. Further exploration of these mechanisms may facilitate the development of more effective immunotherapeutic strategies that further harness the role of the humoral immune system in antitumor immunity.

A shortcoming of our study was small pretreatment biopsy sizes, restricting comprehensive immune tumor microenvironment analysis and allowing only bulk sequencing before ICB. Low cell and patient numbers from single-cell sequencing, limited clonal tracking to unique CDR3 regions and unverified antigen specificity of clonally expanded PCs were additional constraints. For instance, in the radiation plus cemiplimab (PD-1) cohort, PCs were abundant in non-responders but lacked clear IgG1 signatures seen in responders. However, we aimed to overcome these limitations by integrating multiple approaches and cohorts that consistently linked increased IgG1 PC phenotype to posttreatment clinical response.

## Methods

### Cohort descriptions

Discovery cohort (D1). Early-stage HCC lesions and matched non-involved liver specimens were surgically resected after two doses of cemiplimab (ClinicalTrials.gov registration: NCT03916627; cohort B1) or 2−4 doses of nivolumab. Patients across all HCC etiologies responded to ICB, defined as ≥50% tumor necrosis by pathological examination^18^.

Validation cohort (V2). Early-stage HCC lesions and matched non-involved liver specimens were treated with stereotactic body radiotherapy ((SBRT) 8 Gy × three fractions) followed by two doses of cemiplimab prior to surgery. These patients were subsequently surgically resected after two doses of cemiplimab. Patients across all HCC etiologies responded to ICB, defined as ≥50% tumor necrosis by pathological examination^18^ (NCT03916627; cohort B2).

Biopsies and tumor tissues from D1 and V2 cohorts were obtained from these patients undergoing surgical resection at Mount Sinai Hospital, after obtaining informed consent in accordance with a protocol reviewed and approved by the institutional review board (IRB) at the Icahn School of Medicine at Mount Sinai (IRB 18-00407).

Validation cohort (V3), Sade-Feldman et al.^3^. Patients with metastatic melanoma provided written informed consent for the collection of tissue and blood samples for research and genomic profiling, as approved by the Dana-Farber/Harvard Cancer Center IRB (DF/HCC protocol 11-181) and The University of Texas MD Anderson Cancer Center (IRB LAB00-063 and 2012-0846). Tumor samples (*n* = 48) were obtained from 32 patients at baseline and/or after checkpoint therapy. Checkpoint blockade therapy used antibodies targeting CTLA-4, PD-1 or PDL-1 (database of Genotypes and Phenotypes (dbGaP) study accession numbers phs001680.v1.p1 and PRJNA489548).

Validation cohort (V4), IMbrave150 (ref. ^20^): a phase 3, open-label, randomized study of atezolizumab in combination with bevacizumab compared to sorafenib in patients and untreated locally advanced or metastatic HCC. This study evaluated the efficacy and safety of atezolizumab in combination with bevacizumab compared to sorafenib in participants with locally advanced or metastatic HCC who have received no prior systemic treatment. The participants were randomized in a 2:1 ratio to one of the two treatment arms: arm A (experimental arm): atezolizumab + bevacizumab; arm B (control arm): sorafenib (NCT03434379).

Validation cohort (V5), Cappuyns et al.^21^. This cohort was from the University Hospitals Leuven in Leuven, Belgium. Single-cell transcriptomics was used to characterize the intratumoral and peripheral immune context of patients with advanced HCC treated with atezolizumab + bevacizumab. Both blood and tumor tissue were evaluated (EGAS00001007547).

Validation cohort (V6), Ma et al.^22^. This cohort consists of individuals aged 18 years or older diagnosed with gastrointestinal cancers, including throat, stomach, gallbladder, liver, pancreatic or colon cancer, who are scheduled for treatment at the National Institutes of Health (NIH) Clinical Center. Participants will undergo a screening process involving a physical examination and medical history, provide a baseline blood sample and contribute additional blood samples at 2 months and 4 months after baseline as well as at the completion of their treatment, across 1−4 NIH visits. They will also provide tumor tissue samples if they undergo cancer-related surgery, with no treatment provided as part of this study, which focuses on analyzing their immune system’s response to the cancer through these samples. The data are available at the Sequence Read Archive (SRA) repository: GSE151530 (NCT01313442).

Validation cohort (V7), Zhang et al.^60^. This cohort consists of data from the tumor microenvironment in HCC resection specimens from a prospective clinical trial of neoadjuvant cabozantinib, a multi-tyrosine kinase inhibitor that primarily blocks VEGFR2, and nivolumab, a PD-1 inhibitor in which five out of 15 patients were found to have a pathologic response at the time of resection. However, only four responders and three non-responders had data available. The data are available at the SRA repository: GSE238264 (NCT03299946).

Validation cohort (V8)^25–27^. Multiple cohorts with available overall survival data were evaluated with survival analysis. These cohorts include TCGA cohorts^25^. Data were accessed via the Genomic Data Commons (https://portal.gdc.cancer.gov) and https://www.cancer.gov/tcga. Furthermore, we also investigated the cohorts POPLAR and OAK from NCT01903993 and NCT02008227, respectively.

#### Grand serology ELISA

ELISA was used to detect and quantify circulating IgG antibodies to known tumor antigens, as previously described. In brief, plasma samples were analyzed by low-volume semiautomated ELISA for seroreactivity to a panel of recombinant protein antigens (NY-ESO-1, p53, SOX2, HORMAD1, ERG, DHFR, PRAME, WT1, MELAN-A, SURVIVIN, UBTD2, CT47, MAGE-A1, MAGE-A4, SSX4, CT10, SSX2, XAGE, GAGE7 and MAGE-A10). Low-volume 96-well plates were coated overnight at 4 °C with 0.5–1 μg ml^−1^ antigen and blocked for 2 h at room temperature with PBS containing 5% non-fat milk and 0.1% Tween 20. Plasma was titrated from 1:100 to 1:6,400 in fourfold dilutions and added to blocked and washed 96-well plates. For assay validation and titer calculation, each plate contained positive and negative controls (pool of healthy donor sera). After overnight incubation, plates were extensively washed with PBS 0.2% Tween 20 and rinsed with PBS. Plasma antigen-specific IgG was detected after incubation with alkaline-phosphatase-conjugated goat anti-human IgG (SouthernBiotech, 2040-4, diluted 1:4,500), revelation using AttoPhos substrate and buffer and measurement using a fluorescence reader (BioTek Synergy). By linear regression, a reciprocal titer was calculated for each sample and for each antigen as the predicted or interpolated dilution value at which the titration curve meets a cutoff value^7^. A positive significant result was defined as reciprocal titers more than 100.

#### ELISpot assay

After bead-guided selection, CD8^+^ T cells were independently cultured with peptide-pulsed, irradiated T-cell-depleted PBMCs (serving as antigen-presenting cells) in RPMI + 10% serum type AB (to avoid potential reactivity) supplemented with IL-2 (10 U ml^−1^) and IL-7 (20 ng ml^−1^) twice a week. Cells were assessed for specificity at day 10 of culture for CD8, using autologous antigen-presenting cells pulsed with NY-ESO-1 peptides or controls (influenza nucleoprotein peptide pool or dimethyl sulfoxide (DMSO)). The IFNγ ELISpot assay was performed on CD8^+^ T cells. In brief, 96-well nitrocellulose ELISpot plates (Millipore, MAHA S4510) were coated overnight at 4 °C with 2 μg ml^−1^ anti-human IFNγ monoclonal antibody (1-D1K) and blocked with 10% human AB serum containing RPMI 1640 for 2 h at 37 °C. Then, 2 × 10^4^ sensitized CD8^+^ T cells and 2 × 10^4^ peptide-pulsed T-APCs were placed in each well of the ELISpot plate at a final volume of 100 μl of RPMI 1640 medium without serum. After incubation for 22 h at 37 °C in a CO_2_ incubator, the plate was developed using 0.2 μg ml^−1^ biotinylated anti-human IFNγ monoclonal antibody (Mabtech, 7-B6-1), 1 μg ml^−1^ streptavidin-alkaline phosphatase conjugate (Roche Diagnostics) and 5-bromo-4-chloro-3-indolyl phosphate/NBT (Sigma-Aldrich). The number of spots was evaluated using a CTL ImmunoSpot analyzer and software (Cellular Technology Limited). Results are shown as the number of spot-forming cells without subtracting the number of background spots, because the number of spot-forming cells in negative control was fewer than three spots per well in all assays. A positive response with more than 50 spot counts per well as well as spot counts ≥twofold more than background spots obtained with non-pulsed target cells was considered to be significant. The significance was defined descriptively only, if the number of spots observed for NY-ESO-1 in pre, post or lymph node was greater than >2× the number of spots in control DMSO as well as more than 50 spots per 50,000 cells.

#### Seromics

Seromics profiling was performed using CDI Labs’ HuProt Human Proteome Microarray version 4.0, which includes over 21,000 individually purified full-length human proteins and isoforms, providing comprehensive coverage of more than 80% of the human proteome. The proteins were printed in duplicate pairs on PATH nitrocellulose slides (CDI Labs). Patient sera were diluted 1:500 in Seromics Sample Buffer. Simultaneously, the barcoded HuProt Microarrays were blocked using CDIArrayBlock buffer to minimize non-specific binding. The diluted sera samples were applied to the blocked microarrays and incubated for 1 h at room temperature on a shaker. After incubation, the microarrays underwent a series of washes. Goat Anti-Human IgG Fc Cross-Adsorbed Secondary Antibody DyLight 550 and Goat Anti-Human IgA Chain Alpha Antibody DyLight 650 were applied to the microarrays. These were incubated for 1 h at room temperature on a shaker, followed by additional washes. The microarrays were gently dried and scanned immediately using a GenePix 4300A Microarray Scanner, using GenePix Pro software (Molecular Devices). The resulting images were analyzed with Mapix microarray image acquisition and analysis software (Innopsys), where signal intensities of background and positive and negative control spots were quantified.

#### Single-cell RNA-seq

Sample preparation: single-cell suspensions from HCC tissues were obtained, as described above. Cell dissociation was achieved using gentleMACS standard dissociation protocol. Samples were broadly enriched for CD45^+^ cells by fluorescence-activated cell sorting, and these cells were suspended in PBS supplemented with 0.05% BSA. Viability of single cells was assessed using Acridine Orange/Propidium Iodide viability staining reagent (Nexcelom Bioscience), and debris-free suspensions of more than 80% viability were deemed suitable for the experiments. Single-cell RNA-seq was performed using the Chromium platform (10x Genomics) with the 5′ gene expression (5′ GEX) V2 kit, as per the manufacturer’s instructions, for a target cell recovery of 10,000 cells per lane. Both gene expression and BCR V(D)J libraries were constructed, according to the manufacturer’s instructions. All libraries were quantified via Agilent 2100 hsDNA Bioanalyzer or TapeStation 4200 and KAPA library quantification kit (Roche, 0797014001). Libraries were sequenced at a targeted depth of 25,000 reads per cell for gene expression and 5,000 reads per cell for BCR V(D)J, using the paired-end Illumina NovaSeq S4 300-cycle kit.

#### Spatial transcriptomics analysis

Seven HCC samples (cohort V7) profiled using 10x Genomics Visium were integrated using scVI (version 1.3.0). Spatial clustering was performed with CellCharter (version 0.3.3), and differential gene expression analysis was conducted within the scVI framework. For each cluster, canonical immune and HCC-related genes were subjected to pathway enrichment analysis (using gseapy version 1.1.8) to guide annotation. Non-canonical genes were included if uniquely or highly expressed within a specific cluster. Heatmaps of enriched pathways and marker expression profiles were generated for visual comparison.

#### mIHC and TLS community detection

Seventeen mIHC biopsies (six responders and 11 non-responders) from the discovery cohort (D1) were analyzed to identify TLS-like communities. CD3^+^, CD8^+^ and CD20^+^ cells were used as seeds for community detection. Cells within 10 µm of each other were iteratively connected if positive for any of the three markers, forming spatially contiguous TLS-like communities. Radial density profiles were computed for CD20^+^, CD3^+^, CD8^+^ and MZB1^+^ cells within each community by defining concentric rings from the centroid—determined via mean shift clustering—to encompass approximately 10% of the effective community area per ring. Marker-specific densities were calculated cumulatively across these rings.

#### PC infiltration scoring

To assess global infiltration of MZB1^+^ PCs, spatial graphs were constructed using all cell centroids within each tissue section. A *k*-nearest neighbor (KNN) graph (*k* = 10) was constructed and partitioned using the Leiden algorithm. The infiltration score for each community was defined as the ratio of MZB1^+^ cells to the total number of cells within the community. These scores were used to compare PC dispersion between responders and non-responders.

#### Statistical analysis

Gene expression reads were aligned to the hg38 reference transcriptome, and count matrices were generated using the default Cell Ranger 2.1 workflow, using the ‘raw’ matrix output. After alignment, barcodes matching cells contained more than 200 unique genes and at maximum 1,000 counts. From these cells, those with transcripts more than 25% mitochondrial genes were filtered from downstream analyses. Matrix scaling, logarithmic normalization and batch correction via data alignment through canonical correlation analysis and unsupervised clustering using a KNN graph partitioning approach were performed as previously described. Single-cell clustering was done on the top 2,000−3,000 genes based on the dataset. Immunoglobulin light, heavy and variable chains were excluded from clustering due to their overabundance in PCs and B cells. Differentially expressed genes were identified using the FindMarkers function (Seurat). Mean unique molecular identifiers (UMIs) were imputed to determine logarithmic fold changes in expression between cell states to further the analysis of markers of interest. Gene set enrichment analysis was performed using the Enrichr, Gene Ontology and Kyoto Encyclopedia of Genes and Genomes databases. Other R packages used include scDissector version 1.0.0, ComplexHeatmap version 2.0, ggplot2 version 3.3.5, tidyverse version 1.0, Matrix version 0.9.8, seriation version 1.3.5, Dream version 1.0, singleR version 1.0, CellChat version 1.0, Dirichlet version 0.9 and immunarch version 1.5. Survival analyses were performed using the survival, survminer and gtsummary R packages. Differential abundance was done using Dirichlet regression modeling strategies. BCR analysis was done using immunarch and Wilcoxon rank test. Reconstruction of BCRs from bulk and single cells was done using TRUST4 and MixCR algorithms. Trajectory analyses were conducted using Monocle 3 and Moran’s *I* index.

### Reporting summary

Further information on research design is available in the Nature Portfolio Reporting Summary linked to this article.

## Online content

Any methods, additional references, Nature Portfolio reporting summaries, source data, extended data, supplementary information, acknowledgements, peer review information; details of author contributions and competing interests; and statements of data and code availability are available at 10.1038/s41591-025-04177-6.

## Supplementary information

Reporting Summary

Supplemental Table 1 Supplementary File 1 containing the quantification of autoantibodies and statistical results for the seromics assay.

Supplementary Table 2 Supplementary File 2 containing pages for clone tracking and gene signatures per cluster.

## Data Availability

The following external bulk and single-cell RNA-seq datasets were used for analyses shown in this study: GSE206325, GSE238264, GSE120575, GSE151530 and EGAS00001007547. The data generated by this study are available via Zenodo (10.5281/zenodo.17393774)^61^. For additional details, please contact edgar.gonzalez-kozlova@mssm.edu, and we will respond within 48 h.

## Code availability

The analysis code is available at https://github.com/eegk/B_and_Plasma_Cell_Studies. For additional details, please contact edgar.gonzalez-kozlova@mssm.edu, and we will respond within 48 h.

## References

[1] Wei, S. C., Duffy, C. R. & Allison, J. P. Fundamental mechanisms of immune checkpoint blockade therapy. *Cancer Discov.* **8**, 1069–1086 (2018). 30115704 10.1158/2159-8290.CD-18-0367

[2] Laumont, C. M., Banville, A. C., Gilardi, M., Hollern, D. P. & Nelson, B. H. Tumour-infiltrating B cells: immunological mechanisms, clinical impact and therapeutic opportunities. *Nat. Rev. Cancer* **22**, 414–430 (2022). 35393541 10.1038/s41568-022-00466-1 PMC9678336

[3] Sade-Feldman, M. et al. Defining T cell states associated with response to checkpoint immunotherapy in melanoma. *Cell* **175**, 998–1013 (2018). 30388456 10.1016/j.cell.2018.10.038 PMC6641984

[4] Wouters, M. C. A. & Nelson, B. H. Prognostic significance of tumor-infiltrating B cells and plasma cells in human cancer. *Clin. Cancer Res.* **24**, 6125–6135 (2018). 30049748 10.1158/1078-0432.CCR-18-1481

[5] Sui, H. et al. Immunotherapy of targeting MDSCs in tumor microenvironment. *Front. Immunol.* **13**, 990463 (2022). 36131911 10.3389/fimmu.2022.990463 PMC9484521

[6] Magen, A. et al. Intratumoral dendritic cell−CD4^+^ T helper cell niches enable CD8^+^ T cell differentiation following PD-1 blockade in hepatocellular carcinoma. *Nat. Med.* **29**, 1389–1399 (2023). 37322116 10.1038/s41591-023-02345-0 PMC11027932

[7] Laumont, C. M. & Nelson, B. H. B cells in the tumor microenvironment: multi-faceted organizers, regulators, and effectors of anti-tumor immunity. *Cancer Cell* **41**, 466–489 (2023). 36917951 10.1016/j.ccell.2023.02.017

[8] Helmink, B. A. et al. B cells and tertiary lymphoid structures promote immunotherapy response. *Nature* **577**, 549–555 (2020). 31942075 10.1038/s41586-019-1922-8 PMC8762581

[9] Petitprez, F. et al. B cells are associated with survival and immunotherapy response in sarcoma. *Nature* **577**, 556–560 (2020). 31942077 10.1038/s41586-019-1906-8

[10] Griss, J. et al. B cells sustain inflammation and predict response to immune checkpoint blockade in human melanoma. *Nat. Commun.* **10**, 4186 (2019). 31519915 10.1038/s41467-019-12160-2 PMC6744450

[11] Patil, N. S. et al. Intratumoral plasma cells predict outcomes to PD-L1 blockade in non-small cell lung cancer. *Cancer Cell* **40**, 289–300 (2022). 35216676 10.1016/j.ccell.2022.02.002

[12] Fridman, W. H. et al. Tertiary lymphoid structures and B cells: an intratumoral immunity cycle. *Immunity* **56**, 2254–2269 (2023). 37699391 10.1016/j.immuni.2023.08.009

[13] Petroni, G., Pillozzi, S. & Antonuzzo, L. Exploiting tertiary lymphoid structures to stimulate antitumor immunity and improve immunotherapy efficacy. *Cancer Res.* **84**, 1199–1209 (2024). 38381540 10.1158/0008-5472.CAN-23-3325 PMC11016894

[14] Fridman, W. H. et al. B cells and tertiary lymphoid structures as determinants of tumour immune contexture and clinical outcome. *Nat. Rev. Clin. Oncol.* **19**, 441–457 (2022). 35365796 10.1038/s41571-022-00619-z

[15] Yuan, J. et al. CTLA-4 blockade enhances polyfunctional NY-ESO-1 specific T cell responses in metastatic melanoma patients with clinical benefit. *Proc. Natl Acad. Sci. USA* **105**, 20410–20415 (2008). 19074257 10.1073/pnas.0810114105 PMC2629307

[16] Yuan, J. et al. Integrated NY-ESO-1 antibody and CD8^+^ T-cell responses correlate with clinical benefit in advanced melanoma patients treated with ipilimumab. *Proc. Natl Acad. Sci. USA* **108**, 16723–16728 (2011). 21933959 10.1073/pnas.1110814108 PMC3189057

[17] Germain, C. et al. Presence of B cells in tertiary lymphoid structures is associated with a protective immunity in patients with lung cancer. *Am. J. Respir. Crit. Care Med.* **189**, 832–844 (2014). 24484236 10.1164/rccm.201309-1611OC

[18] Marron, T. U. et al. Neoadjuvant cemiplimab for resectable hepatocellular carcinoma: a single-arm, open-label, phase 2 trial. *Lancet Gastroenterol. Hepatol.* **7**, 219–229 (2022). 35065058 10.1016/S2468-1253(21)00385-X PMC9901534

[19] Buckup, M. et al. Multiparametric cellular and spatial organization in cancer tissue lesions with a streamlined pipeline. *Nat. Biomed. Eng*. 10.1038/s41551-025-01475-9 (2025). 10.1038/s41551-025-01475-9 PMC13008776 40855123

[20] Kudo, M. et al. IMbrave150: efficacy and safety of atezolizumab plus bevacizumab versus sorafenib in patients with Barcelona Clinic Liver Cancer stage B unresectable hepatocellular carcinoma: an exploratory analysis of the phase III study. *Liver Cancer.* **12**, 238–250 (2022). 37767068 10.1159/000528272 PMC10521324

[21] Cappuyns, S. et al. PD-1^−^CD45RA^+^ effector-memory CD8 T cells and CXCL10^+^ macrophages are associated with response to atezolizumab plus bevacizumab in advanced hepatocellular carcinoma. *Nat. Commun.* **14**, 7825 (2023). 38030622 10.1038/s41467-023-43381-1 PMC10687033

[22] Ma, L. et al. Tumor cell biodiversity drives microenvironmental reprogramming in liver cancer. *Cancer Cell* **36**, 418–430 (2019). 31588021 10.1016/j.ccell.2019.08.007 PMC6801104

[23] Matsuo, M. et al. IFN-γ enables cross-presentation of exogenous protein antigen in human Langerhans cells by potentiating maturation. *Proc. Natl Acad. Sci. USA* **101**, 14467–14472 (2004). 15383663 10.1073/pnas.0405947101 PMC521945

[24] Gupta, A. et al. A novel human-derived antibody against NY-ESO-1 improves the efficacy of chemotherapy. *Cancer Immun.* **13**, 3 (2013). 23390374 PMC3559191

[25] Cancer Genome Atlas Research Network et al. The Cancer Genome Atlas Pan-Cancer analysis project. *Nat. Genet.* **45**, 1113–1120 (2013). 24071849 10.1038/ng.2764 PMC3919969

[26] Fehrenbacher, L. et al. Atezolizumab versus docetaxel for patients with previously treated non-small-cell lung cancer (POPLAR): a multicentre, open-label, phase 2 randomised controlled trial. *Lancet* **387**, 1837–1846 (2016). 26970723 10.1016/S0140-6736(16)00587-0

[27] Rittmeyer, A. et al. Atezolizumab versus docetaxel in patients with previously treated non-small-cell lung cancer (OAK): a phase 3, open-label, multicentre randomised controlled trial. *Lancet* **389**, 255–265 (2017). 27979383 10.1016/S0140-6736(16)32517-X PMC6886121

[28] Nutt, S. L., Hodgkin, P. D., Tarlinton, D. M. & Corcoran, L. M. The generation of antibody-secreting plasma cells. *Nat. Rev. Immunol.* **15**, 160–171 (2015). 25698678 10.1038/nri3795

[29] Morgan, D. & Tergaonkar, V. Unraveling B cell trajectories at single cell resolution. *Trends Immunol.* **43**, 210–229 (2022). 35090788 10.1016/j.it.2022.01.003

[30] Yang, B. et al. An Asia-specific variant of human IgG1 represses colorectal tumorigenesis by shaping the tumor microenvironment.*J. Clin. Invest.* **132**, e153454 (2022). 35133976 10.1172/JCI153454 PMC8920342

[31] Gu, Y. et al. Tumor-educated B cells selectively promote breast cancer lymph node metastasis by HSPA4-targeting IgG. *Nat. Med.* **25**, 312–322 (2019). 30643287 10.1038/s41591-018-0309-y

[32] Leader, A. M. et al. Single-cell analysis of human non-small cell lung cancer lesions refines tumor classification and patient stratification. *Cancer Cell* **39**, 1594–1609 (2021). 34767762 10.1016/j.ccell.2021.10.009 PMC8728963

[33] Iwata, Y. et al. Characterization of a rare IL-10–competent B-cell subset in humans that parallels mouse regulatory B10 cells. *Blood* **117**, 530–541 (2011). 20962324 10.1182/blood-2010-07-294249 PMC3031478

[34] Ng, K. W. et al. Antibodies against endogenous retroviruses promote lung cancer immunotherapy. *Nature* **616**, 563–573 (2023). 37046094 10.1038/s41586-023-05771-9 PMC10115647

[35] Lavie, D., Ben-Shmuel, A., Erez, N. & Scherz-Shouval, R. Cancer-associated fibroblasts in the single-cell era. *Nat. Cancer* **3**, 793–807 (2022). 35883004 10.1038/s43018-022-00411-z PMC7613625

[36] Aranda, C. J. et al. IgG memory B cells expressing *IL4R* and *FCER2* are associated with atopic diseases. *Allergy* **78**, 752–766 (2023). 36445014 10.1111/all.15601 PMC9991991

[37] Mlynarczyk, C. et al. *BTG1* mutation yields supercompetitive B cells primed for malignant transformation. *Science* **379**, eabj7412 (2023). 36656933 10.1126/science.abj7412 PMC10515739

[38] Calame, K. L., Lin, K.-I. & Tunyaplin, C. Regulatory mechanisms that determine the development and function of plasma cells. *Annu. Rev. Immunol.* **21**, 205–230 (2003). 12524387 10.1146/annurev.immunol.21.120601.141138

[39] Kim, S. S. et al. B cells improve overall survival in HPV-associated squamous cell carcinomas and are activated by radiation and PD-1 blockade. *Clin. Cancer Res.* **26**, 3345–3359 (2020). 32193227 10.1158/1078-0432.CCR-19-3211 PMC7334097

[40] Kang, W. et al. Tertiary lymphoid structures in cancer: the double-edged sword role in antitumor immunity and potential therapeutic induction strategies. *Front. Immunol.* **12**, 689270 (2021). 34394083 10.3389/fimmu.2021.689270 PMC8358404

[41] Schumacher, T. N. & Thommen, D. S. Tertiary lymphoid structures in cancer. *Science* **375**, eabf9419 (2022). 34990248 10.1126/science.abf9419

[42] Roudko, V. et al. Immunological biomarkers of response and resistance to treatment with cabozantinib and nivolumab in recurrent endometrial cancer. *J. Immunother. Cancer* **13**, e010541 (2025). 40010771 10.1136/jitc-2024-010541 PMC11865721

[43] Ohue, Y. et al. Serum antibody against NY-ESO-1 and XAGE1 antigens potentially predicts clinical responses to anti-programmed cell death-1 therapy in NSCLC. *J. Thorac. Oncol.* **14**, 2071–2083 (2019). 31449889 10.1016/j.jtho.2019.08.008

[44] Wang, H. et al. NY-ESO-1 expression in solid tumors predicts prognosis: a systematic review and meta-analysis. *Medicine (Baltimore)* **98**, e17990 (2019). 31770209 10.1097/MD.0000000000017990 PMC6890322

[45] Gure, A. O. et al. Cancer-testis genes are coordinately expressed and are markers of poor outcome in non-small cell lung cancer. *Clin. Cancer Res.* **11**, 8055–8062 (2005). 16299236 10.1158/1078-0432.CCR-05-1203

[46] Velazquez, E. F. et al. Expression of the cancer/testis antigen NY-ESO-1 in primary and metastatic malignant melanoma (MM)–correlation with prognostic factors. *Cancer Immun.* **7**, 11 (2007). 17625806 PMC2935749

[47] Dai, Y. et al. Humoral determinants of checkpoint immunotherapy. *Nature* **644**, 527–536 (2025). 40702172 10.1038/s41586-025-09188-4

[48] Hao, D. et al. The single-cell immunogenomic landscape of B and plasma cells in early-stage lung adenocarcinoma. *Cancer Discov.* **12**, 2626–2645 (2022). 36098652 10.1158/2159-8290.CD-21-1658 PMC9633381

[49] Wu, R.-Q. et al. Immune checkpoint therapy-elicited sialylation of IgG antibodies impairs antitumorigenic type I interferon responses in hepatocellular carcinoma. *Immunity* **56**, 180–192 (2023). 36563676 10.1016/j.immuni.2022.11.014

[50] Luan, X. et al. Cyclophilin A is a key positive and negative feedback regulator within interleukin-6 trans-signaling pathway. *FASEB J.* **35**, e21958 (2021). 34606626 10.1096/fj.202101044RRR

[51] Xie, Y., Li, X. & Ge, J. STAT3−CyPA signaling pathway in endothelial cell apoptosis. *Cell. Signal.* **65**, 109413 (2020). 31494257 10.1016/j.cellsig.2019.109413

[52] Calandra, T. & Roger, T. Macrophage migration inhibitory factor: a regulator of innate immunity. *Nat. Rev. Immunol.* **3**, 791–800 (2003). 14502271 10.1038/nri1200 PMC7097468

[53] Tang, C.-H. A. et al. Phosphorylation of IRE1 at S729 regulates RIDD in B cells and antibody production after immunization. *J. Cell Biol.* **217**, 1739–1755 (2018). 29511123 10.1083/jcb.201709137 PMC5940306

[54] Bujisic, B. et al. Impairment of both IRE1 expression and XBP1 activation is a hallmark of GCB DLBCL and contributes to tumor growth. *Blood* **129**, 2420–2428 (2017). 28167662 10.1182/blood-2016-09-741348

[55] Zhu, H., Jiang, C., Kaufman, R. J., Li, H. & Singh, N. In vitro stimulation of IRE1α/XBP1-deficient B cells with LPS. *Methods Mol. Biol.* **2378**, 221–231 (2022). 34985703 10.1007/978-1-0716-1732-8_14 PMC9382655

[56] Franiak-Pietryga, I. et al. Activated B cells and plasma cells are resistant to radiation therapy. *Int. J. Radiat. Oncol. Biol. Phys.* **112**, 514–528 (2022). 34474108 10.1016/j.ijrobp.2021.08.037 PMC8976465

[57] Long, F. et al. The potential crosstalk between tumor and plasma cells and its association with clinical outcome and immunotherapy response in bladder cancer. *J. Transl. Med.* **21**, 298 (2023). 37138324 10.1186/s12967-023-04151-1 PMC10155334

[58] Altorki, N. K. et al. Neoadjuvant durvalumab plus radiation versus durvalumab alone in stages I−III non-small cell lung cancer: survival outcomes and molecular correlates of a randomized phase II trial. *Nat. Commun.* **14**, 8435 (2023). 38114518 10.1038/s41467-023-44195-x PMC10730562

[59] Ban, Y. et al. Radiation-activated secretory proteins of *Scgb1a1*^+^ club cells increase the efficacy of immune checkpoint blockade in lung cancer. *Nat. Cancer* **2**, 919–931 (2021). 34917944 10.1038/s43018-021-00245-1 PMC8670735

[60] Zhang, S. et al. Spatial transcriptomics analysis of neoadjuvant cabozantinib and nivolumab in advanced hepatocellular carcinoma identifies independent mechanisms of resistance and recurrence. *Genome Med.* **15**, 72 (2023). 37723590 10.1186/s13073-023-01218-y PMC10506285

[61] Gonzalez-Kozlova, E. (ed.) Humoral IgG1 responses to tumor antigens underpin clinical outcomes in immune checkpoint blockade. *Zenodo*10.5281/zenodo.17393774 (2025). 10.1038/s41591-025-04177-6 PMC13004670 41593194

