# Supplementary material for: Humoral IgG1 responses to tumor antigens underpin clinical outcomes in immune checkpoint blockade
Source: Nat Med. 2026 Jan 27;32(3):978–91. doi: 10.1038/s41591-025-04177-6 (PMC13004670; doi:10.1038/s41591-025-04177-6)
Supplement: Supplementary file 1 — Reporting Summary [file 41591_2025_4177_MOESM1_ESM.pdf]

nature portfolio

Corresponding author(s): Sacha Gnjatic

Last updated by author(s): Sep 17, 2025

Reporting Summary

Nature Portfolio wishes to improve the reproducibility of the work that we publish. This form provides structure for consistency and transparency in reporting. For further information on Nature Portfolio policies, see our Editorial Policies and the Editorial Policy Checklist.

Statistics

For all statistical analyses, confirm that the following items are present in the figure legend, table legend, main text, or Methods section.

n/a	Confirmed	
☐	☒	The exact sample size (*n*) for each experimental group/condition, given as a discrete number and unit of measurement
☐	☒	A statement on whether measurements were taken from distinct samples or whether the same sample was measured repeatedly
☐	☒	The statistical test(s) used AND whether they are one- or two-sided<br>*Only common tests should be described solely by name; describe more complex techniques in the Methods section.*
☐	☒	A description of all covariates tested
☐	☒	A description of any assumptions or corrections, such as tests of normality and adjustment for multiple comparisons
☐	☒	A full description of the statistical parameters including central tendency (e.g. means) or other basic estimates (e.g. regression coefficient) AND variation (e.g. standard deviation) or associated estimates of uncertainty (e.g. confidence intervals)
☐	☒	For null hypothesis testing, the test statistic (e.g. *F*, *t*, *r*) with confidence intervals, effect sizes, degrees of freedom and *P* value noted<br>*Give P values as exact values whenever suitable.*
☒	☐	For Bayesian analysis, information on the choice of priors and Markov chain Monte Carlo settings
☒	☐	For hierarchical and complex designs, identification of the appropriate level for tests and full reporting of outcomes
☐	☒	Estimates of effect sizes (e.g. Cohen's *d*, Pearson's *r*), indicating how they were calculated

Our web collection on statistics for biologists contains articles on many of the points above.

Software and code

Policy information about availability of computer code

Data collection

```
Tools and R packages used: R 4.2.0,  dendextend_1.19.0       lubridate_1.9.4        forcats_1.0.0
##  [4] stringr_1.5.1          purrr_1.0.4            readr_2.1.5
##  [7] tidyr_1.3.1            tidyverse_2.0.0        ggbeeswarm_0.7.2
## [10] viridis_0.6.5          viridisLite_0.4.2      data.table_1.17.0
## [13] cowplot_1.1.3          Vennerable_3.1.0.9000  colorspace_2.1-1
## [16] pals_1.10              anndata_0.7.5.6        ggdendro_0.2.0
## [19] RColorBrewer_1.1-3     tibble_3.2.1           scales_1.4.0
## [22] factoextra_1.0.7       ComplexHeatmap_2.20.0  doParallel_1.0.17
## [25] iterators_1.0.14       foreach_1.5.2          clustree_0.5.1
## [28] ggraph_2.2.1           ggsci_3.2.0            ggrepel_0.9.6
## [31] swimplot_1.2.0         circlize_0.4.16        rms_8.0-0
## [34] Hmisc_5.2-3            rmarkdown_2.29         dplyr_1.1.4
## [37] cccd_1.6               igraph_2.1.4           Rtsne_0.17
## [40] mclust_6.1.1           tsne_0.1-3.1           pheatmap_1.0.12
## [43] reshape2_1.4.4         reshape_0.8.9          matrixStats_1.5.0
## [46] forestmodel_0.6.2      survminer_0.5.0        ggpubr_0.6.0
## [49] survival_3.8-3         DirichletReg_0.7-1     Formula_1.2-5
## [52] variancePartition_1.35.5 BiocParallel_1.38.0  limma_3.60.6
## [55] crumblr_0.99.11        ggplot2_3.5.2
##
## loaded via a namespace (and not attached):
```

```
##   [1] fs_1.6.6                   bitops_1.0-9
##   [3] httr_1.4.7                 numDeriv_2016.8-1.1
##   [5] tools_4.4.1                backports_1.5.0
##   [7] R6_2.6.1                   lazyeval_0.2.2
##   [9] GetoptLong_1.0.5           litedown_0.7
##  [11] withr_3.0.2                gridExtra_2.3
##  [13] quantreg_6.1               cli_3.6.5
##  [15] Biobase_2.64.0             gt_1.0.0
##  [17] sandwich_3.1-1             labeling_0.4.3
##  [19] sass_0.4.10                mvtnorm_1.3-3
##  [21] survMisc_0.5.6             polspline_1.1.25
##  [23] proxy_0.4-27               commonmark_1.9.5
##  [25] yulab.utils_0.2.0          foreign_0.8-90
##  [27] dichromat_2.0-0.1          labelled_2.14.0
##  [29] maps_3.4.2.1               rstudioapi_0.17.1
##  [31] FNN_1.1.4.1                generics_0.1.3
##  [33] gridGraphics_0.5-1         shape_1.4.6.1
##  [35] gtools_3.9.5               car_3.1-3
##  [37] Matrix_1.7-3               S4Vectors_0.42.1
##  [39] abind_1.4-8                lifecycle_1.0.4
##  [41] multcomp_1.4-28            yaml_2.3.10
##  [43] gtsummary_2.2.0            carData_3.0-5
##  [45] SummarizedExperiment_1.34.0 pamr_1.57
##  [47] gplots_3.2.0               SparseArray_1.4.8
##  [49] crayon_1.5.3               lattice_0.22-7
##  [51] haven_2.5.4                mapproj_1.2.11
##  [53] pillar_1.10.2              knitr_1.50
##  [55] GenomicRanges_1.56.2       tcltk_4.4.1
##  [57] rjson_0.2.23               boot_1.3-31
##  [59] corpcor_1.6.10             codetools_0.2-20
##  [61] glue_1.8.0                 ggfun_0.1.8
##  [63] broom.helpers_1.20.0       vctrs_0.6.5
##  [65] png_0.1-8                  treeio_1.28.0
##  [67] Rdpack_2.6.4               gtable_0.3.6
##  [69] assertthat_0.2.1           cachem_1.1.0
##  [71] zigg_0.0.2                 xfun_0.52
##  [73] rbibutils_2.3              S4Arrays_1.4.1
##  [75] Rfast_2.1.5.1              tidygraph_1.3.1
##  [77] reformulas_0.4.0           SingleCellExperiment_1.26.0
##  [79] KMsurv_0.1-5               maxLik_1.5-2.1
##  [81] statmod_1.5.0              TH.data_1.1-3
##  [83] nlme_3.1-168               pbkrtest_0.5.3
##  [85] ggtree_3.12.0              EnvStats_3.0.0
##  [87] GenomeInfoDb_1.40.1        bslib_0.9.0
##  [89] vipor_0.4.7                KernSmooth_2.23-26
##  [91] rpart_4.1.24               BiocGenerics_0.50.0
##  [93] nnet_7.3-20                tidyselect_1.2.1
##  [95] compiler_4.4.1             graph_1.82.0
##  [97] htmlTable_2.4.3            SparseM_1.84-2
##  [99] xml2_1.3.8                 DelayedArray_0.30.1
## [101] tcltk2_1.2-11              checkmate_2.3.2
## [103] caTools_1.18.3             remaCor_0.0.18
## [105] RBGL_1.80.0                digest_0.6.37
## [107] minqa_1.2.8                aod_1.3.3
## [109] XVector_0.44.0             RhpcBLASctl_0.23-42
## [111] htmltools_0.5.8.1          pkgconfig_2.0.3
## [113] base64enc_0.1-3            lme4_1.1-37
## [115] MatrixGenerics_1.16.0      fastmap_1.2.0
## [117] rlang_1.1.6                GlobalOptions_0.1.2
## [119] htmlwidgets_1.6.4          UCSC.utils_1.0.0
## [121] farver_2.1.2               jquerylib_0.1.4
## [123] zoo_1.8-14                 jsonlite_2.0.0
## [125] magrittr_2.0.3             GenomeInfoDbData_1.2.12
## [127] ggplotify_0.1.2            patchwork_1.3.0
## [129] Rcpp_1.0.14                ape_5.8-1
## [131] reticulate_1.42.0          stringi_1.8.7
## [133] zlibbioc_1.50.0            MASS_7.3-65
## [135] plyr_1.8.9                 deldir_2.0-4
## [137] graphlayouts_1.2.2         splines_4.4.1
## [139] hms_1.1.3                  markdown_2.0
## [141] ggsignif_0.6.4             stats4_4.4.1
## [143] evaluate_1.0.3             RcppParallel_5.1.10
## [145] tzdb_0.5.0                 nloptr_2.2.1
## [147] tweenr_2.0.3               cards_0.6.0
## [149] miscTools_0.6-28           MatrixModels_0.5-4
## [151] polyclip_1.10-7            km.ci_0.5-6
```

```
## [153] clue_0.3-66          ggforce_0.4.2
## [155] broom_1.0.8            xtable_1.8-4
## [157] fANCOVA_0.6-1            e1071_1.7-16
## [159] tidytree_0.4.6          rstatix_0.7.2
## [161] class_7.3-23           stylo_0.7.5
## [163] lmerTest_3.1-3           aplot_0.2.5
## [165] beeswarm_0.4.0             memoise_2.0.1
## [167] IRanges_2.38.1           cluster_2.1.8.1
## [169] timechange_0.3.0
```

Data analysis

All software used is freely available. Code Availability
The analysis code is available under https://github.com/eegk/B_and_Plasma_Cell_Studies. For additional details please contact edgar.gonzalez-kozlova@mssm.edu and we will respond within 48 hours.

For manuscripts utilizing custom algorithms or software that are central to the research but not yet described in published literature, software must be made available to editors and reviewers. We strongly encourage code deposition in a community repository (e.g. GitHub). See the Nature Portfolio guidelines for submitting code & software for further information.

Data

Policy information about availability of data

All manuscripts must include a data availability statement. This statement should provide the following information, where applicable:

- Accession codes, unique identifiers, or web links for publicly available datasets
- A description of any restrictions on data availability
- For clinical datasets or third party data, please ensure that the statement adheres to our policy

Data Availability
Sequencing Datasets. The following external bulk and single-cell RNA sequencing datasets were used for analyses shown in this study: GSE206325 (https://www.ncbi.nlm.nih.gov/geo/query/acc.cgi?acc=GSE206325) , GSE238264 (https://www.ncbi.nlm.nih.gov/geo/query/acc.cgi?acc=GSE238264), GSE120575 (https://www.ncbi.nlm.nih.gov/geo/query/acc.cgi?acc=GSE120575), GSE151530 (https://www.ncbi.nlm.nih.gov/geo/query/acc.cgi?acc=GSE151530), and EGAS00001007547 (https://ega-archive.org/studies/EGAS00001007547). The data generated by this study is available Zenodo (doi:10.5281/zenodo.17393774 or https://zenodo.org/records/17393774). For additional details please contact edgar.gonzalez-kozlova@mssm.edu and we will respond within 48 hours.
Code Availability
The analysis code is available under https://github.com/eegk/B_and_Plasma_Cell_Studies. For additional details please contact edgar.gonzalez-kozlova@mssm.edu and we will respond within 48 hours.

Research involving human participants, their data, or biological material

Policy information about studies with human participants or human data. See also policy information about sex, gender (identity/presentation), and sexual orientation and race, ethnicity and racism.

Reporting on sex and gender

Sex was considered as a covariate for all differential expression analyses intially, however, no sex-associated differences were observed in any of the analyzed outcomes. This is consistent with the balanced representation of male and female patients in the cohort.

Reporting on race, ethnicity, or other socially relevant groupings

Race information was not included in the analyses as it did not account for sufficient amount of variance explained to justify the inclusion, and is not correlated with the tested measures.

Population characteristics

Detailed information at each clinical trial NCT number provided in the recruitment section.

Recruitment

Discovery cohort (D1). Early-stage HCC lesions and matched noninvolved liver specimens that were surgically resected after two doses of cemiplimab (ClinicalTrials.gov registration https://clinicaltrials.gov/study/NCT03916627, cohort B1) or two to four doses of nivolumab. Patients across all HCC etiologies responded to ICB, defined as ≥50% tumor necrosis by pathological examination (45).
Validation cohort (V2). Early-stage HCC lesions and matched noninvolved liver specimens that were treated with stereotactic body radiotherapy (SBRT 8 Gy X 3 fractions) followed by two doses of cemiplimab prior to surgery. These patients were subsequently surgically resected after two doses of cemiplimab. Patients across all HCC etiologies responded to ICB, defined as ≥50%tumor necrosis by pathological examination (45). (https://clinicaltrials.gov/study/NCT03916627, cohort B2).
Validation cohort (V3) Sade-Feldman et al. Patients with metastatic melanoma provided written informed consent for the collection of tissue and blood samples for research and genomic profiling, as approved by the Dana-Farber/Harvard Cancer Center Institutional Review Board (DF/HCC Protocol 11-181) and UT MD Anderson Cancer Center (IRB LAB00-063 and 2012-0846). Tumor samples (n=48) were obtained from 32 patients at baseline and/or after checkpoint therapy. Checkpoint blockade therapy using antibodies targeting CTLA4, PD1 or PDL1. dbGaP Study Accession: phs001680.v1.p1, PRJNA489548.
Validation cohort (V4) IMbrave150. A Phase III, Open-Label, Randomized Study of Atezolizumab in Combination With Bevacizumab Compared With Sorafenib in Patients and Untreated Locally Advanced or Metastatic Hepatocellular Carcinoma. This study evaluated the efficacy and safety of atezolizumab in combination with bevacizumab compared with sorafenib in participants with locally advanced or metastatic Hepatocellular Carcinoma (HCC) who have received no prior systemic treatment. The participants were randomized in a 2:1 ratio to one of the two treatment arms: Arm A (experimental arm): Atezolizumab +bevacizumab; Arm B (control arm): Sorafenib. (https://clinicaltrials.gov/study/NCT03434379)
Validation Cohort (V5) Cappuyns et al., Cohort from the University Hospitals Leuven, Leuven, Belgium. Single-cell

transcriptomics was used to characterize the intra-tumoral and peripheral immune context of patients with advanced HCC treated with atezo/bev. Both Blood and Tumor tissue was evaluated. (https://www.ega-archive.org/studies/EGAS00001007547)
Validation Cohort (V6) Ma et al., This cohort consists of individuals aged 18 or older diagnosed with gastrointestinal cancers, including throat, stomach, gallbladder, liver, pancreatic, or colon cancer, who are scheduled for treatment at the National Institutes of Health (NIH) Clinical Center. Participants will undergo a screening process involving a physical examination and medical history, provide a baseline blood sample, and contribute additional blood samples at 2 and 4 months post-baseline, as well as at the completion of their treatment, across one to four NIH visits. They will also provide tumor tissue samples if they undergo cancer-related surgery, with no treatment provided as part of this study, which focuses on analyzing their immune system's response to the cancer through these samples. The data is available at SRA repository GSE151530 (https://clinicaltrials.gov/study/NCT01313442).
Validation Cohort (V7) Zhang et al. This cohort consists of data from the tumor microenvironment in HCC resection specimens from a prospective clinical trial of neoadjuvant cabozantinib, a multi-tyrosine kinase inhibitor that primarily blocks VEGFR2, and nivolumab, a PD-1 inhibitor in which 5 out of 15 patients were found to have a pathologic response at the time of resection. However, only 4 responders and 3 non-responders had data available. The data is available at SRA repository GSE238264 (https://clinicaltrials.gov/study/NCT03299946).
Validation cohort (V8). Multiple cohorts with available survival OS data were evaluated with survival analysis. These cohorts include The Cancer Genome Atlas (TCGA) cohorts, Weinstein et al., 2013: "The Cancer Genome Atlas Pan-Cancer analysis project." Nature Genetics, 45(10):1113–20. DOI: 10.1038/ng.2764. Data were accessed via the Genomic Data Commons (GDC) (https://portal.gdc.cancer.gov) and https://www.cancer.gov/tcga. Further, we also investigated the cohorts POPLAR and OAK from https://clinicaltrials.gov/study/NCT01903993 and https://clinicaltrials.gov/study/NCT02008227 , respectively.

The following external bulk and single-cell RNA sequencing datasets were used for analyses shown in this study: GSE206325, GSE238264, GSE120575, GSE151530, and EGAS00001007547. Additional sequencing data and code is available upon request.

Ethics oversight

The study was performed under IRB-approved guidance and regulations to keep all patient information strictly de-identified.

Note that full information on the approval of the study protocol must also be provided in the manuscript.

Field-specific reporting

Please select the one below that is the best fit for your research. If you are not sure, read the appropriate sections before making your selection.

- [x] Life sciences
- [ ] Behavioural & social sciences
- [ ] Ecological, evolutionary & environmental sciences

For a reference copy of the document with all sections, see nature.com/documents/nr-reporting-summary-flat.pdf

Life sciences study design

All studies must disclose on these points even when the disclosure is negative.

Sample size

Cohorts include D1 (n=38), V2 (n=10), V3 (n=48), V4 (358), V5 (n=38), V6 (n=46), V7 (n=7), V8: TCGA-SKCM (n= 380), POPLAR (n=192), OAK (699), TCGA-LUSC (n=241), TCGA-LIHC (n=70)

Data exclusions

We used the available data from each clinical trial and cohort described above. We did not exclude any data point from analysis.

Replication

We compared the findings of our study with seven different sets of cohorts available from SRA and Array Express. The IDs of these studies are provided in the methods section.

Randomization

Our cohorts were randomized for seamless integration and avoidance of batch specific effects. Further, data integration methods were used to correct for technical artifacts. Statistical analysis using mixed effect models were applied as needed to adjust for technical effects. Cohort-wise, Phase 3 clinical trials were randomized while smaller cohorts were not randomized for covariates.

Blinding

Unsupervised methods were used for clustering which are blinded to clinical outcomes. No additional blinding was used.

Reporting for specific materials, systems and methods

We require information from authors about some types of materials, experimental systems and methods used in many studies. Here, indicate whether each material, system or method listed is relevant to your study. If you are not sure if a list item applies to your research, read the appropriate section before selecting a response.

Materials & experimental systems

n/a	Involved in the study
☐	☒ Antibodies
☒	☐ Eukaryotic cell lines
☒	☐ Palaeontology and archaeology
☒	☐ Animals and other organisms
☒	☐ Clinical data
☒	☐ Dual use research of concern
☒	☐ Plants

Methods

n/a	Involved in the study
☒	☐ ChIP-seq
☒	☐ Flow cytometry
☒	☐ MRI-based neuroimaging

Antibodies

Antibodies used

GST-HT-ESO1 (Nishikawa) 1.63mg/ml 4/4/14
NY-ESO-1 protein 0.5mg/ml IDCc002 8/17/15 MIA(IMP) 13581 Inmuno Design
GST-HT-P53 1.24mg/ml 2/17/12
GST-HT-MAGE-A1 4mg/ml 3/9/12
GST-HT-MAGE-A3 Baculo 294 ug/ml 01/18/02
GST-HT-MAGE-A4 4.6mg/ml 10/27/11
GST-HT-MAGE-A10 3.9mg.ml 2/17/12
GST-HT-SOX2 0.28 mg/ml 2014/3/20
SSX2 6.5 mg/ml 2012/2/28
GST-HT-SSX4 1.7mg/ml 2/28/12
GST-HT-CT10 3.72mg/ml 4/14/14
GST-HT-CT47 0.96mg/ml 2/17/12
MELAN-A 3.2mg/ml 4M Urea ph7.5 4/22/2005 ITH
GST-HT-HORMAD1 0.72mg/ml 3/18/14
GST-HT-SURVIVIN Δ EX3 1.0mg/ml 2/8/13
HERV-K 0.5mg/ml 11/19/2014
GST-HT-UBTD2 0.65mg/ml in glycerol 6/29/12
GST-HT-XAGE 2.901mg/ml 9/8/14
XAGE1b 2.823mg/ml
WT1 Ag BMP319 0.79mg/ml Exp. 01/06/17
ASCI a-PRAME Lot:P1206F Prep.22Jun12 0.57mg/ml (GSK Vaccines)
GST-HT-ERG 2.665mg/ml 9/4/14
GST-HT-GAGE7 2.22mg/ml 8/7/14
DHFR- 0.196 mg/mL 2017/02/07
NY-ESO-1 (LICR) protein 0.5mg/ml Manu: 7/23/15 Lot: PBR-0039-001-LICR
and CDI labs HuProt protein arrays.

Validation

References:
Gnjatic, S., Old, L. J., & Chen, Y. T. (2009). Autoantibodies against cancer antigens. Methods Mol Biol, 520, 11-19. Retrieved from https://www.ncbi.nlm.nih.gov/pubmed/19381944. doi:10.1007/978-1-60327-811-9_2

Manufacturer website: https://www.cdilabs.com/products/huprot-microarray

Plants

Seed stocks

NA

Novel plant genotypes

NA

Authentication

NA
